# Androgen excess: a hallmark of polycystic ovary syndrome

**DOI:** 10.3389/fendo.2023.1273542

**Published:** 2023-12-13

**Authors:** Kexin Wang, Yanhua Li, Yu Chen

**Affiliations:** ^1^ The Second School of Clinical Medicine, Zhejiang Chinese Medical University, Hangzhou, China; ^2^ Department of General Practice, The Second Affiliated Hospital of Zhejiang Chinese Medical University, Hangzhou, China

**Keywords:** androgen receptor, Hyperandrogenism, insulin resistance, polycystic ovarian syndrome, steroidogenesis

## Abstract

Polycystic ovarian syndrome (PCOS) is a metabolic, reproductive, and psychological disorder affecting 6–20% of reproductive women worldwide. However, there is still no cure for PCOS, and current treatments primarily alleviate its symptoms due to a poor understanding of its etiology. Compelling evidence suggests that hyperandrogenism is not just a primary feature of PCOS. Instead, it may be a causative factor for this condition. Thus, figuring out the mechanisms of androgen synthesis, conversion, and metabolism is relatively important. Traditionally, studies of androgen excess have largely focused on classical androgen, but in recent years, adrenal-derived 11-oxygenated androgen has also garnered interest. Herein, this Review aims to investigate the origins of androgen excess, androgen synthesis, how androgen receptor (AR) signaling mediates adverse PCOS traits, and the role of 11-oxygenated androgen in the pathophysiology of PCOS. In addition, it provides therapeutic strategies targeting hyperandrogenism in PCOS.

## 1 Introduction

Polycystic ovary syndrome (PCOS) is an endocrine, metabolic, reproductive, and psychological disorder affecting approximately 6%–20% of reproductive women worldwide, regardless of ethnicity. It is primarily characterized by ovulation dysfunction, clinical or biochemical hyperandrogenemia (HA), and polycystic ovarian morphology (PCOM) (1). According to the Rotterdam criteria, two of the three abovementioned features are required for the diagnosis of PCOS. Although this standard is widely accepted, the definition of PCOS by the National Institutes of Health is mainly focused on two aspects: HA and ovulatory dysfunction. As for the Androgen Excess Society criteria, the presence of HA is required along with either ovulatory dysfunction or PCOM or both. Hence, four PCOS phenotypes (A, B, C, and D) are ascertained under the Rotterdam criteria. Patients should be thoroughly assessed to exclude other conditions that cause symptoms similar to PCOS. Although approximately 75% of patients with PCOS exhibit insulin resistance (IR), IR is not recognized as a diagnostic criterion (2).

**Figure 1:**
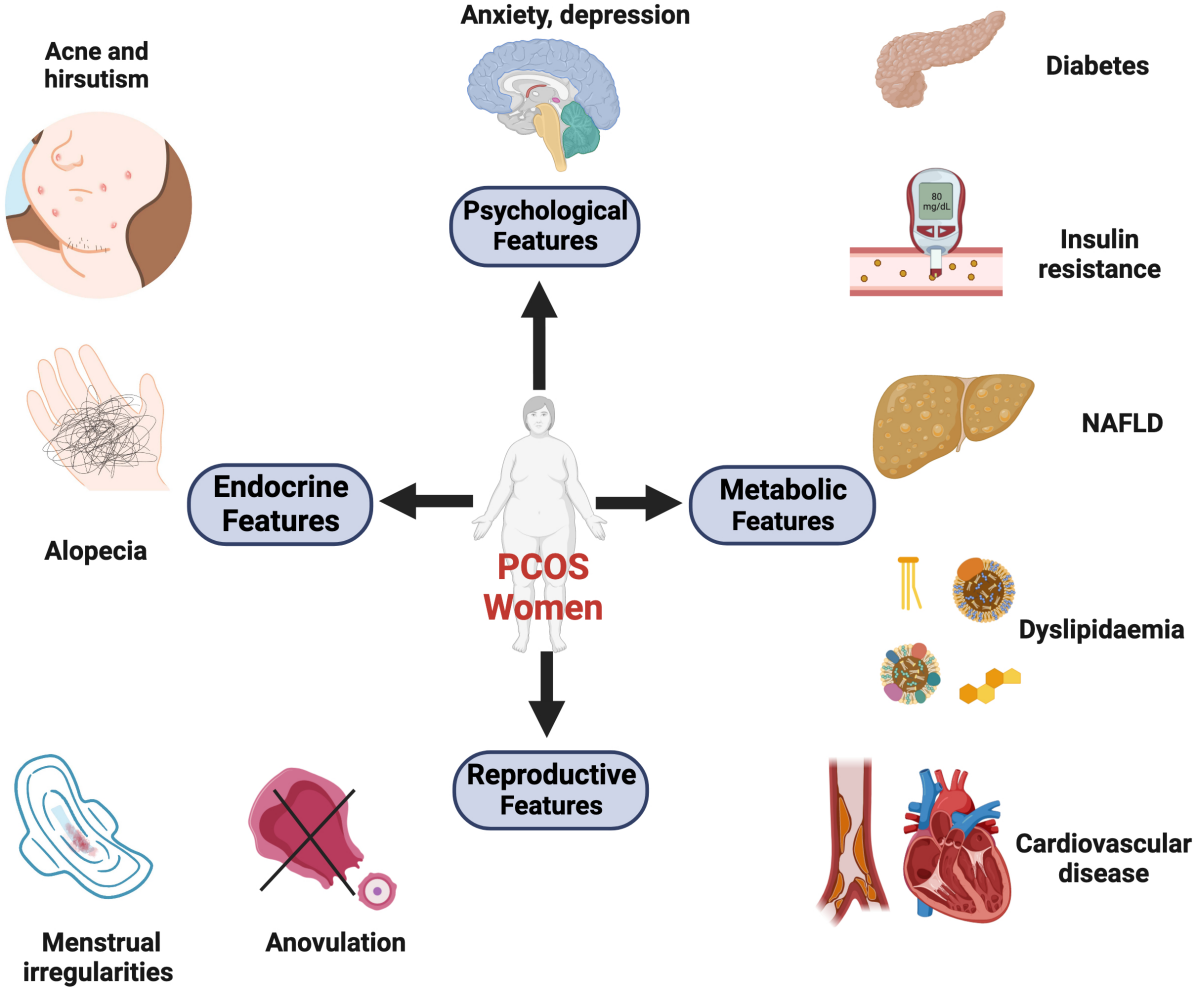
The major clinical manifestations of PCOS. These symptoms can be divided into four categories: reproductive, endocrine, metabolic, and psychological comorbidities. (1) Reproductive features: the dysregulated hypothalamus-pituitary-gonadal (HPG) axis and neuroendocrine factors contribute to menstrual irregularities, anovulation, infertility, and increased risks of pregnancy complications. (2) Endocrine features: hormonal imbalances (ovarian and adrenal hyperandrogenemia). Symptoms like hirsutism, acne, and androgenic alopecia are induced by high levels of circulating androgens. (3) Metabolic features: 30% of lean and 70% of obese patients exhibit insulin resistance. Additionally, women with PCOS suffer from abdominal obesity, dyslipidemia, and non-alcoholic fatty liver disease (NAFLD). These metabolic abnormalities can result in long-term cardiometabolic sequelae, including type 2 diabetes, hypertension, and atherosclerotic disease. (4) Psychological features: PCOS is associated with an increased prevalence of depression, anxiety, and poor quality of life. Created with BioRender.com.

As PCOS is a highly heterogeneous disease, its exact cause remains unknown. Abundant evidence suggests that environment, prenatal, and genetic factors, as well as epigenetic changes, are interrelated with each other (3). Patients with PCOS may still experience poor metabolic outcomes regardless of whether their body mass index (BMI) is within the normal range. A previous study has demonstrated that the incidence of non-alcoholic fatty liver disease is correlated with HA, after adjusting for BMI (4). Furthermore, patients with PCOS with HA are more likely to develop type 2 diabetes later in life than those with normal androgen levels (5). Additionally, HA exposure leads to a higher prevalence of cardiometabolic complications, such as hypertension and obesity. This could be explained by the activation of the renin–angiotensin system, deregulation of sympathetic nervous system activity, and upregulation of androgen receptors (ARs) (6). Moreover, excess maternal androgen exposure can impair the placental function, increase the risk of developing PCOS in female offspring, and reduce sperm quality in male generations (7) (**Figure 1**).

Under the Rotterdam classification, patients with phenotype D do not exhibit HA but only symptoms of oligo-anovulation and ultrasonographic evidence of PCOM. One study reviewed several recent publications and surprisingly determined that there was no evident difference in metabolic parameters between patients with phenotype D and controls, although some of these normoandrogenic patients from East Asian populations present with higher metabolic risks (8). Moreover, androgen levels decline dramatically with age in patients with phenotype D PCOS, and even decrease below the average levels of their healthy counterparts when they reach ≥35 years. Gleicher et al. redefined this phenotype as the hyper-/hypoandrogenic phenotype (HH-PCOS). This phenotype may be driven by immune dysregulation and adrenal autoimmunity, indicating a different etiopathology than the other three phenotypes (9). Therefore, the application of the Rotterdam criteria has been controversial, and an alternative classification for the diagnosis of PCOS has been proposed in recent years (10). Overall, more individualized and tailored treatments targeting different PCOS phenotypes are required.

Considering that the majority (60%–80%) of women with PCOS have elevated androgen levels, we focused on the interactions between IR and HA, synthesis of androgen in target organs and tissues, action of androgen through ARs, and promising therapeutic interventions to relieve hyperandrogenic symptoms.

## 2 Insulin resistance

IR and hyperandrogenism are often interconnected and collectively account for the reproductive and metabolic characteristics of PCOS. It is widely argued that IR and subsequent compensatory hyperinsulinemia in PCOS are caused by post-receptor defects, which manifest as decreased tyrosine phosphorylation and increased serine phosphorylation of insulin receptors and insulin receptor substrate (IRS) (11). Hyperinsulinemia inhibits the secretion of the hepatic sex hormone-binding protein (SHBG), thereby increasing free testosterone (T) levels in circulation. Additionally, insulin and luteinizing hormone (LH) administration independently and synergistically stimulates androgen and progesterone production by upregulating the activities of 17-hydroxylase/17,20-lyase (CYP17) and steroidogenic acute regulatory protein (StAR) in ovarian theca cells (TCs). Moreover, insulin and human chorionic gonadotropin combination treatment upregulates CYP11A1 (P450scc) production in TCs (12). Additionally, acute insulin stimulation promotes adrenal steroidogenesis by amplifying the response to ACTH stimulation, with increased 5α-reductase enzyme activity (13). Moreover, one meta-analysis further concluded the increased peripheral 5α-reductase activity in women with PCOS, and it is associated with IR, unrelated to BMI (14).

Insulin signaling pathways are divided into two categories: phosphatidylinositol-3-kinase/serine/threonine-specific protein kinase B (PI3K/AKT), which is related to metabolism, and mitogen-activated protein kinase (MEK)-extracellular signal-regulated kinase (ERK), which is involved in cell growth and proliferation. A growing body of evidence has demonstrated that the aberrant PI3K/AKT pathway in patients with PCOS results in reduced translocation of glucose transporter 4 (GLUT4) from adipocyte and endometrial cells, thus impairing glucose uptake. Decreased endometrial *Glut4* gene expression is also regulated by ARs through binding to the GLUT promoter in rat models (15). Interestingly, one three-dimensional culture of mouse follicles *in vitro* demonstrated that high concentrations of insulin-like growth factor-1 (IGF-1) could inhibit mouse follicular development and maturation, which provides a possible explanation for the involvement of IGF-1 in the pathogenesis of ovulatory disorders in phenotype D in the absence of HA (2, 16). Moreover, enhanced CYP17 activity in TCs is regulated by the PI3K/AKT pathway (17). Despite disrupted PI3K/AKT signaling, the mitogenic insulin receptor-activated pathway remains intact or is enhanced in skin fibroblasts and skeletal muscle from patients with PCOS, which is called selective IR (18, 19).

## 3 Neuroendrocine abnormalities

The hypothalamic–pituitary–gonadal axis regulates ovarian steroidogenesis. Negative feedback from progesterone and estradiol to the hypothalamus maintains sex hormone levels within the normal range. In women with PCOS, increased GnRH pulsatility stimulates greater LH secretion than follicle-stimulating hormone (FSH), thereby increasing the LH-to-FSH ratio. Consequently, it promotes LH-stimulated androgen production in ovarian TCs. Androgen is required for follicular growth in the early phases; however, excess androgen suppresses the expression of cumulus expansion-related genes and oocyte maturation-related genes, causing excess small growing follicles to be arrested at the antral stage, inhibiting the development of the dominant follicle (20). HA in PCOS also disrupts the preovulatory LH surge. Altogether, these findings suggest anovulation in patients with PCOS.

Ovarian anti-Müllerian hormone (AMH) is primarily synthesized by granulosa cells (GCs) from preantral and small antral follicles with a maximum diameter of 8 mm due to the inhibitory effect of E2. Therefore, AMH levels were 2–3 times higher in the serum and follicular fluid of patients with PCOS than in normal women (21). AMH inhibits the sensitivity of growing follicles to FSH. Moreover, E2 suppressed AMH and AMH-specific type 2 receptor (AMHR2) expression at the ovarian level (21). Excess dihydrotestosterone (DHT) directly increases AMH, whereas T upregulates ERα expression through the conversion to E2. High AMH levels activate downstream inhibitory SMAD-6/7 signaling and result in follicular arrest (22). Elevated T levels have a greater correlation with increased AHM levels than other types of androgens (23). Considering the close relationship between HA and AMH, AMH is used to evaluate the degree of HA and is considered an alternative to diagnosing PCOS, although the cutoff values are inconsistent (24). Additionally, AMH has been reported to stimulate GnRH and LH release; however, how AMH modulates GnRH function remains largely unknown. Recently, one study uncovered the role of AMH in regulating GnRH neuron activity in a murine model (25). The structure of the hypothalamic median eminence is altered after AMH binds to AMH2R; thus, the retracted tanycytes make it easier for GnRH neuron terminals to release GnRH into the blood capillaries.

Interestingly, central progesterone or estradiol receptors are not present in GnRH neurons in humans (26). Therefore, upstream KNDy neurons may collectively regulate the activity of GnRH neurons. KNDy neurons distributed in the arcuate nucleus (ARN) are composed of kisspeptin, neurokinin B (NKB), and dynorphin A. Kisspeptin and NKB stimulate kisspeptin release, which is considered a major GnRH pulse generator. Dynorphin A inhibits GnRH release.

HA has been reported to stimulate KNDy neurons, embodied by increased kisspeptin and NKB expression and downregulated DynA expression in rats, thus causing a vicious cycle of hypothalamic–pituitary–ovarian (HPO) dysfunction (27). Treatment with anti-epileptic drugs, which increases the gamma-aminobutyric acid (GABA) concentration in the brain, increases the occurrence of PCOS. This supports the notion that dysfunctional GABA neurons also participate in regulating GnRH neuronal activity (28). The chronic activation of GABA neurons induced PCOS-like symptoms in healthy female rodents, causing elevated T levels and an impaired reproductive cycle. Prenatally androgenized (PNA) mice exhibited attenuated responsiveness to GABA stimulation (29). Mechanistically, when GABA binds to the GABA_b_ receptor expressed in GnRH neurons, it promotes Cl^−^ influx, thus exciting GnRH neurons. Overall, circuit remodeling is evident in GABAergic and GnRH neurons when they are exposed to excess androgens (30).

## 4 Sexual dimorphism

A Mendelian randomization analysis using statistics from the UK Biobank revealed the sex-specific association of testosterone in men and women. A genetically determined 1 standard deviation (SD) higher T level would result in a 15% lower risk of developing type 2 diabetes in men. In accordance with the study, previous research has demonstrated the positive impact of androgen on reducing fat mass in men (31). By contrast, high circulating T levels were detrimental to women. Every 1 SD increase in T levels would increase the occurrence of type 2 diabetes by 37%, and this was associated with a higher PCOS risk with an odds ratio (OR) of 1.51 in women. This study indicated that hyperandrogenism is not simply a feature or consequence but a causative factor in PCOS development (32). Moreover, high androgen levels determined approximately 20% of heritability in both men and women. A metabolomic analysis revealed a similar sexual dimorphism (33). Obesity increases the metabolites of serum branched chains and aromatic amino acids; however, this adverse effect only affects women, with no deleterious effect on men of normal weight or with obesity (33). Numerous studies have demonstrated that women with excess androgen and men with androgen deficiency exhibit overlapping metabolic traits, indicating sexual androgen dimorphism. A good example is PCOS in women and men with hypogonadism (34). Theoretically, females with high estrogen and low androgen levels are prone to gain subcutaneous fat rather than visceral fat and display increased glucose uptake, thus protecting them from adverse metabolic consequences. By contrast, androgens help males have higher muscle mass and reduce abdominal adiposity. Clinical studies have shown that men with androgen deficiency have worse metabolic phenotypes, such as impaired glucose tolerance and higher CVD risks. As for women with PCOS, excess male sex hormones favor abdominal visceral fat deposition, which is also known as the android fat pattern (35).

## 5 Gene polymorphisms

Accumulating evidence indicates that polymorphisms in multiple genes play important roles in PCOS susceptibility and pathogenesis. In recent decades, polymorphisms in several common genes have been identified in the development of PCOS, such as variants in *DEND1A*, *THADA*, *FSHR*, and *LHCGR* gene variants (36). Heidarzadehpilehrood et al. retrieved and summarized the roles of several critical gene mutations involved in steroidogenesis pathways, including *CYP11A1*, *CYP17A1*, and *CYP19A1*, in PCOS pathophysiology. For example, the rs743572 polymorphism in *CYP17* is associated with severe biochemical and clinical characteristics. Allele rs2414096 of the *CYP19* gene is linked to HA and reduced aromatase activity (37). Some findings may be inconsistent among different ethnic populations, and correlations between polymorphisms in the CYP11A1 and CYP17 promoters and T levels in women with PCOS have been reported (38). One study has demonstrated that although polymorphisms in the *AMH* and *AMHR2* genes were not associated with a higher risk of developing PCOS, patients with PCOS with AMH Ile(49)Ser (rs10407022) may represent a milder phenotype due to decreased AMH bioactivity (39). Interestingly, polymorphism rs10406324 (−210 A>G) in the AMH promoter region is associated with lower AMH levels in patients with PCOS (40). Women with a shorter CAG repeat length (≤17) in the *AR* gene may have a higher risk of developing PCOS. In terms of *SHBG*, eight or more *SHBG* gene pentanucleotide TAAAA repeats (rs35785886) have been associated with low serum SHBG levels in women with PCOS (41). The association between *AR* gene polymorphism and polycystic ovary syndrome will be discussed later (in the AR section).

## 6 Androgen synthesis

### 6.1 Androgen synthesis in ovaries

Ovarian androgens are produced by TCs under LH stimulation, whereas estrogen is synthesized in GCs in response to FSH. Owing to the absence of CYP17A1 in GCs, parts of androgens spread from TCs to GCs to produce estrogen. The subtypes of estrogen are determined by different androgen substrates, with A4 converting to estrone (E1) and T aromatizing to estradiol (E2). Moreover, E1 could further be transformed into E2 by 17β-hydroxysteroid dehydrogenase type1 (17β-HSD1). E1 has weaker bioactivity than E2 (42). Unlike the mixed results of E2 levels in classical and non-classical PCOS groups, nearly all patients with PCOS experience increased E1 levels, thus making E1 levels a reliable biomarker for distinguishing between patients with PCOS and healthy women (43, 44).

Androstenedione (A4) is the most abundant serum steroid metabolite in patients with PCOS. Additionally, higher androsterone, T, and 11-ketotestosterone (11-KT) levels and reduced progesterone (PROG/P4) levels were observed when comparing patients with PCOS with healthy matched groups. The synthesis of DHT through the backdoor pathway, which bypasses the formation of T, is enhanced in the ovaries of patients with PCOS. The conversion from 17OH-Prog to 17OH-dihydroprogesterone by 5α-reductase 1 is the starting point of the backdoor pathway, and ultimately, DHT is produced by retinol dehydrogenase and 3β-HSD1/3(AKR1C2/4) from precursor androstanediol in GCs (45).

**Figure 2:**
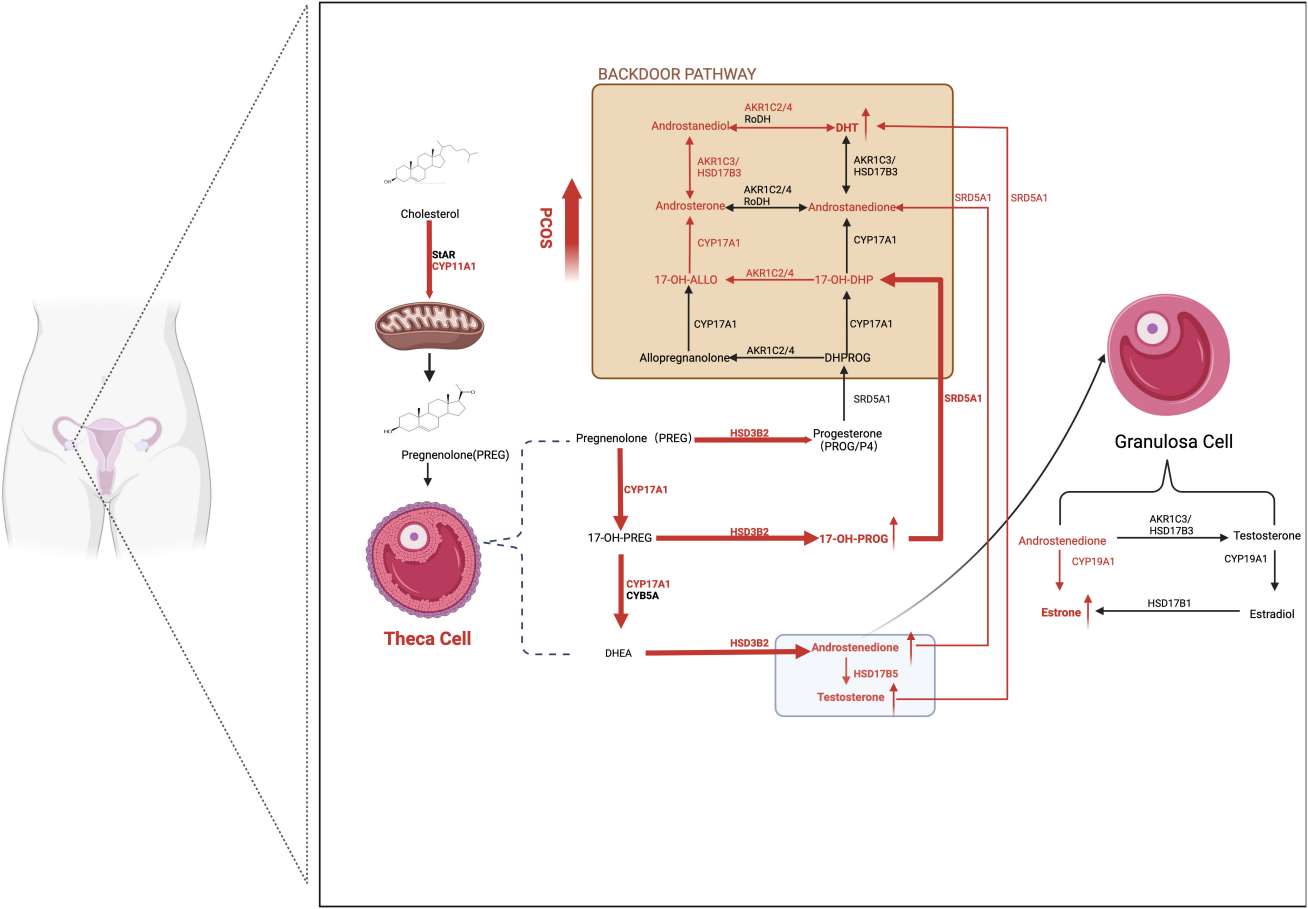
Ovarian-derived androgen biosynthesis in PCOS women (the classical and backdoor pathway). A series of steroidogenic enzymes, including CYP11A1, CYP17A1, and HSD3B2 in the theca cells and CYP19A1 in granulosa cells, are reported to increase in PCOS patients. It is demonstrated that circulating A4 was preferentially elevated in women with PCOS. Furthermore, globally higher SRD5A1 activity is observed, resulting in increased downstream androstanedione and DHT activation from A4 and T, respectively. The backdoor pathway, which involves the production of DHT bypassing the formation from T, is enhanced. In detail, 17OHP4 is the starting point of the backdoor pathway. Because 17OH-Allo is more efficiently metabolized by CYP17A1 than 17OH-Preg, it preferentially synthesizes DHT through the backdoor pathway rather than the classic pathway. The red arrows represent the enhanced pathways. SRD5A1, 5a-reductase type I; A4, androstenedione; T, testosterone; DHT, dihydrotestosterone; 17OHP4, 17ahydroxy-progesterone; 17OH-Allo, 17-hydroxy-allopregnanolone.

Multiple studies have reported that TCs in patients with PCOS are predisposed to generate more androgens due to overactive and overexpressed steroidogenic enzymes, such as CYP17A1, CYP11A1, HSD3B2, SRD5A1, and 17β-HSD5 (also known as AKR1C3) (46). Patients with PCOS with the *DENND1A* splice variant (DENND1A.V2), which encodes the DENND1A protein, demonstrated increased CYP17A1 and CYP11A1 expression and androgen production. Knockout of DENND1A.V2 reduced *CYP17A1* and *CYP11A1* gene transcription and subsequent androgen biosynthesis (47). Moreover, aromatase activity was downregulated in GCs in human polycystic ovaries due to hypomethylation of the CYP19A1 promoter influenced by HA. Additionally, FSH receptors are downregulated in women with PCOS, which may partly explain the lean or non-obese phenotype of PCOS (**Figure 2**).

In bovines, A4 accumulation inhibits GC proliferation and promotes apoptosis, resulting in an arrested cell cycle. Specifically, increased AMH levels induced by HA stimulate CTNNBIP1 expression, which in turn suppresses the interaction of CTNNBIP with Wnt, leading to dysregulated Wnt signaling (48). One study has demonstrated that AR indirectly binds to the promoter of klotho, a regulator of autophagy and aging, embodied with increased klotho levels. Subsequently, this induces the apoptosis of GCs (49). One study disclosed how a hyperandrogenic micro-environment affects a rodent’s ovary. The expression of vascular cell adhesion molecule 1 (Vcam1) was upregulated in TCs and stroma cells identified by dual ARs and NR2F2 expression. Vcam1 is characteristic of Leydig cells and may be linked to immune attachment and inflammation. Interestingly, similar changes were not detected in the GCs (50).

Numerous previous studies have demonstrated that androgen excess disturbs the immune response in the ovary and other targeted organs, with an increased secretion of inflammatory cytokines and dysregulated immune cells (51). Chronic low-grade inflammation, in turn, modulates the expression of steroidogenic genes and initiates a series of physiological processes in theca-interstitial cells (52). For example, IFN-γ expression is inversely associated with dehydroepiandrosterone (DHEA) levels in a dose- and time-dependent manner in GCs (53). DHT treatment increased nuclear AR abundance and thus activated TLR4-IRF-7-NFκB signaling, causing endometrial inflammation (54). One study treated lean and healthy women with DHEA and observed that such treatment stimulates TNFα release by mononuclear cells and AR mRNA in the fasting state. These alterations are positively correlated with increased androgen levels. When pretreated with flutamide, TNFα levels decreased (55).

### 6.2 Androgen synthesis in adipose tissue

Adipose tissues (ATs) are also the main source of androgen production with complete steroidogenic machinery, especially in women with obesity (56). ATs can be classified into two categories: white and brown AT, which are composed of visceral adipose tissue (VAT) and subcutaneous adipose tissue (SAT). These two types of ATs manifest significant characteristics in many aspects. In the SAT of women with PCOS, increased activity of aldo-keto reductase1C1-3 is observed, whereas the enzymes aromatase and 5α-reductase1 are downregulated, indicating increased T biosynthesis and DHT inactivation. Moreover, leptin levels are inversely correlated with DHT concentration (57). These findings are in line with those of a previous study reporting that insulin significantly stimulates A4 and T production in SAT (58). Conversely, in the VAT of women with obesity, increased aromatase activity, representing the hypertrophy of adipocyte, parallels the increased androgen catabolism via enhancing AKR1C2 function (59). In addition, the expression level of AR in the VAT may be higher than that in the SAT of women with PCOS, making the VAT more vulnerable to androgen exposure (35). Hence, in the VAT of women with PCOS, androgen tends to promote lipolysis, whereas insulin-induced lipolysis is suppressed. Thus, excessive free fatty acid efflux leads to ectopic fat deposition, including in the liver and skeletal muscles. Adipocytes become hyperplastic and hypertrophic when exposed to androgen, which triggers immune cell infiltration. Moreover, hyperandrogenism stimulates the transcription of AR in mononuclear cells to release more inflammatory factors (60). Consequently, this stimulates the abnormal secretion of adipocytokines by adipocytes and results in decreased adiponectin production and elevated leptin, visfatin, and resistin levels. The functions of different types of adipocytokines have been described well by various previous articles (61).

Even in normal-weight patients with PCOS, IR is present in AT and positively correlated with serum androgen levels. Androgen modulates the alteration in subcutaneous abdominal adipose stem cell (ASC) gene expression (62). Compared with abdominal SAT, the expression of genes involved in fat accumulation and angiogenesis in gluteofemoral SAT was downregulated. Along with the pro-inflammatory states and low gene expression of adipogenesis in ACSs of gluteofemoral SAT, these findings indicate the restricted adipose expansion in the lower body region. Moreover, the ASCs from gluteofemoral SAT are hypermethylated. The capacity for fat storage may be programmed at the early stages of life, thus promoting the predisposition toward abdominal fat accumulation (63). This opinion is supported by the finding of differential DNA methylation from the VAT of PNA rhesus monkeys (64).

The activity and volume of brown adipose tissue (BAT) are decreased in women with PCOS due to the downregulated expression of uncoupling protein 1 upon excess androgen stimulation, which may cause mitochondrial dysfunction and impaired thermogenesis, which would decrease lipolysis (65). Cold treatment and BAT transplant may be attractive strategies.

### 6.3 Androgen synthesis in the adrenal cortex

The adrenal cortex comprises three layers with different distributions of steroidogenic enzymes. Androgen biosynthesis occurs in the zona reticularis (ZR) (66).

ZR accounts for half of DHEA synthesis, with 20% occurring in the ovary and the remaining 30% being transformed by circulating DHEAS. In contrast to DHEA, its sulfated metabolite, DHEAS, comes exclusively from the adrenal gland, which is attributed to the high and specific expression of sulfotransferase 2A1 in the ZR. Because of the relatively low expression of HSD3B2 in the ZR, the ZR tends to produce DHEAS rather than A4. Thus, its downstream product T is negligible because of the small amount of adrenal HSD17B5. Therefore, DHEAS was used as a reliable marker to assess adrenal androgen excess (67). Genetic variants in the *SULT2A1* gene, for example, SNP rs182420 identified in patients with PCOS, contribute to higher DHEAS levels (68). Interestingly, previous studies have linked low DHEAS concentrations to higher cardiovascular risks and poor lipid profiles in both sexes. DHEAS and DHEA mediate the relaxation of vascular smooth muscles via the mechanism of the openness of potassium channels, thus lowering blood pressure (69). Additionally, high circulating DHEAS helps to lower total cholesterol levels and prevent visceral fat accumulation (70). Similarly, women with PCOS with a high DHEA phenotype have lower carotid intima-media thickness. Moreover, DHEAS decreases with age at similar rates between women with PCOS and their healthy counterparts (71, 72). However, DHEAS measurement cannot accurately reflect the actual amounts of adrenal androgen. As precursors, DHEA and DHEAS can be converted to other forms of androgen in the periphery. Furthermore, women with PCOS may have exaggerated adrenal steroidogenesis in response to ACTH stimulation. One study used a dexamethasone suppression test to distinguish the sources of androgen in women with PCOS. Surprisingly, total testosterone levels decreased dramatically in 9 out of 51 patients after glucocorticoid suppression, indicating adrenal-derived androgen dominance. However, among them, only one patient demonstrated elevated baseline DHEAS levels (73). This study suggested that excess serum dehydroepiandrosterone sulfate does not parallel adrenal hyperandrogenism.

### 6.4 11-oxygenated androgen

Emerging evidence has demonstrated that adrenal-derived 11-oxygenated androgens are also key players in the development of PCOS. 11-oxygenated androgens acquired their name because they share the same oxygen atom on the 11th carbon (74).

Because of the specific expression of CYP11B1 in the ZR, A4, and T serve as substrates and are then converted to 11-OH-androstenedione (11-OH-A4) and 11OH-testosterone (11-OH-T), respectively. 11-OH-A4 is the most abundant 11-oxygenated androgen in the adrenal cortex, considering the minor abundance of T for low HSD17B5 activity. In the kidney, 11β-hydroxysteroid dehydrogenase type 2 (HSD11B2) is responsible for catalyzing 11-OH-A4 and 11-OH-T into 11-ketoandrostenedione (11-KA4) and 11-ketotestosterone (11-KT). Therefore, the majority of circulating 11-KT is converted from 11-KA4 by HSD17B5. Under 5α-reductase stimulation, 11-KT is metabolized to 11-ketodihydrotestosterone (11-KDHT), which has similar bioactivity to DHT. Similarly, 11-KT has equivalent potency to T (75). However, 11β-hydroxy derivatives, including 11-OH-A4, 11-OH-T, and 11-OH-DHT, exhibit low or no androgenic bioactivity in mammalian cells (76). Considering the low affinity of 5α-reductase for 11-KT, serum 11-KDHT only constitutes a minor proportion of the overall circulating androgen pool when compared with DHT (77). The backdoor pathway for 11-oxygenated androgen begins with P4 or 17α-hydroxyprogesterone (17OHP4). After a series of conversions, the end product, 11-KDHT, was obtained (66).

CYP11B1 expression is almost negligible in the ovary. Moreover, 11-oxygenated androgens are unfavorable substrates for the enzyme CYP19A1, which explains why 11-oxygenated estrogen does not exist (78). Consistently, one study measured the levels of 11-KT and its precursors and observed that although 11-KT levels were elevated in patients with PCOS, no distinct difference in 11-KT and 11-OHA4 levels was observed between the ovarian vein and periphery (79). Moreover, the administration of oral contraceptive does not completely block HA in women with PCOS. These findings indicate that the ovary is not the only source of excess androgens in PCOS.

As the 11-oxygenated androgens are predominantly secreted by adrenal glands rather than the ovary, they are therefore not controlled by the HPO axis but by the ACTH. They follow diurnal rhythms similar to cortisol (79). The levels of traditional androgen decline with age, whereas 11-oxygenated androgen levels are quite stable. Owing to the age-related degradation of ZR, the zonal boundary of HSD3B2 and CYB5A becomes less distinct. Hence, promoting the conversion from DHEA into A4 and thus downstream 11-oxygenated androgens through the action of HSD3B2 (80). This may partly explain why women with PCOS still have a higher cardiovascular risk even after menopause (81–83). As for adolescents, there is no evident increase in 11-KT in patients with PCOS, and metformin administration has no effect on 11-KT levels (84). One study compared the 11-oxygenated androgen levels between girls whose mothers have PCOS and obesity and healthy individuals. Unfortunately, no evident difference was observed between the two groups (85). The dominant circulating androgen during the adrenarche is 11-HT, and premature adrenarche may be the possible explanation (86).

Intriguingly, one study identified 11-oxygenated androgens as the predominant circulating androgens in women with PCOS, with 11-KT levels being more than twice as high as T (87). However, androgen levels vary among patients with PCOS (88). Another study supports BMI as a key player in determining steroid profiles (89). The 11-oxygenated androgens are considered to be higher in patients with obesity and PCOS than in healthy counterparts without obesity. They are positively correlated with IR, partly because HSD17B5 upregulates lipid accumulation in the obese group. Dumesic et al. have reported that the normal level of 11-oxygenated androgens in women of normal weight and with PCOS is associated with reduced cortisol levels and decreased HSD11B1 activity. This protective effect diminishes as visceral fat mass increases (90). HSD17B5 (AKR1C3) is induced by the insulin with PI3K-AKT-mTOR signaling (88). Moreover, one study has reported that excess 11-oxygenated androgen production is triggered by heightened adrenal insulin signaling (91). Specifically, insulin directly upregulates steroidogenic factor 1 (SF-1) expression to stimulate the synthesis of adrenal steroidogenic enzymes, including StAR, CCYP11A1, CYP11B1, CYB11B2, and HSD3B2, and suppresses FoxO1 nuclear translocation (92).

**Figure 3:**
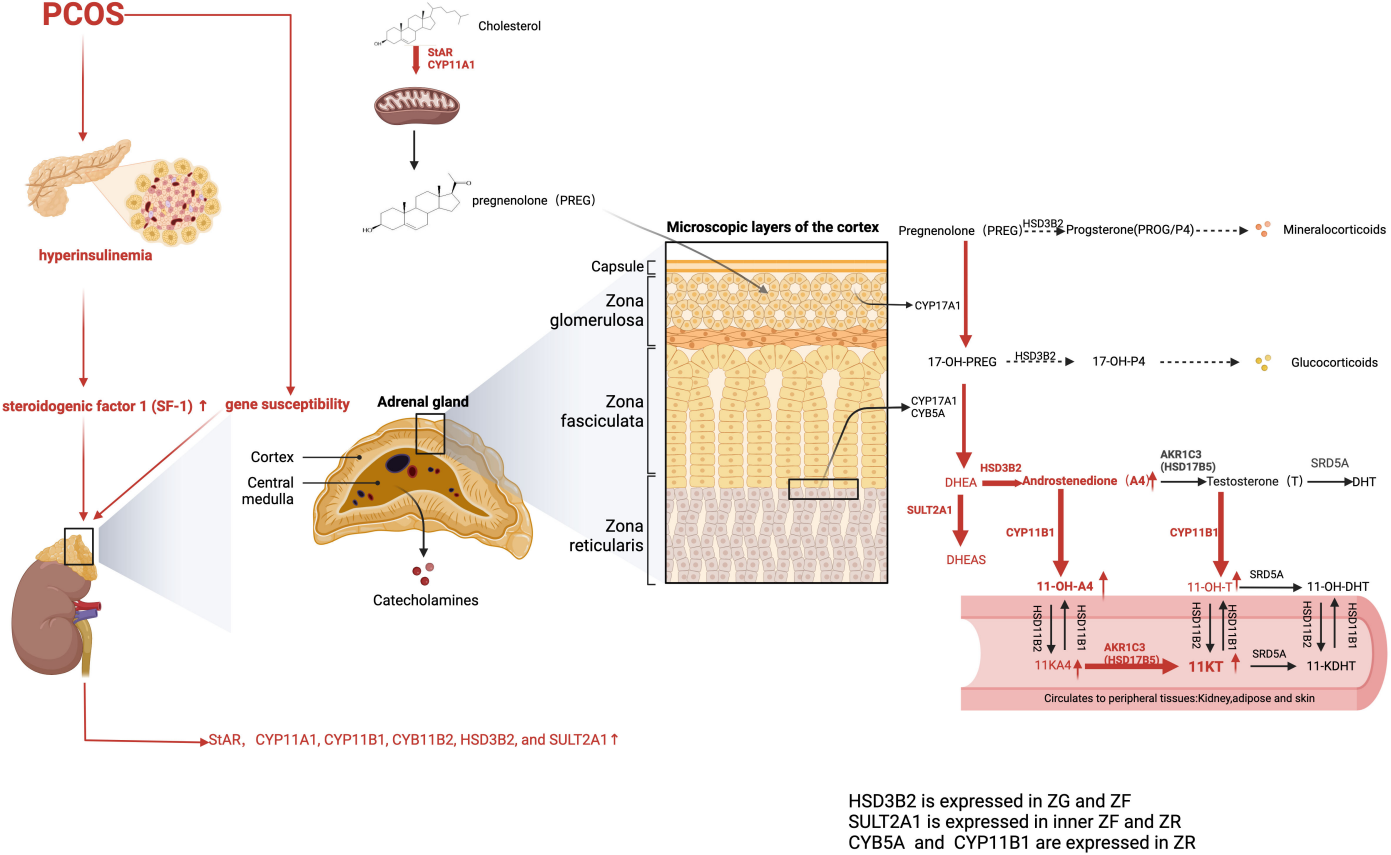
Adrenal-derived androgen synthesis in PCOS women (the classical and backdoor pathway). The levels of adrenal-derived A4, DHEA, and its sulfated metabolite DHEAS were significantly higher in PCOS patients. AKR1C3, also known as HSD17B5, is primarily expressed in peripheral tissues and only minimally distributed in the adrenal gland, which explains the negligible contents of T in the adrenal gland. Hence, 11OHA4 has the highest content of 11-oxygenated androgen content in the adrenal cortex. As for PCOS patients, the 11-oxygenated androgens 11-OHA-4, 11-KA4, 11-OH-T, and 11-KT are all elevated. Moreover, 11-KT is the predominant 11-oxygenated androgen in circulation. The red arrows represent the enhanced pathways. A4, Androstenedione; DHEA, dehydroepiandrosterone; 11-OH-A4, 11-OH-androstenedione; 11-KA4, 11-ketoandrostenedione; 11OH-T, 11OH-testosterone; 11-KT, 11-ketotestosterone.

In addition, HSD17B5 has a higher affinity for 11-KA4 converting into 11-KT than classical androgen. 11-KT is further inactivated and transformed into 11-OH-T in the presence of HSD11B1 (93). Moreover, HSD17B5 has been reported to stabilize and activate AR without androgen binding and to upregulate fatty acid synthase, thus leading to excess lipogenesis and lipotoxicity (94). Therefore, the ratio of HSD17B5 to HSD11B1 plays a crucial role in determining the severity of hyperandrogenism in patients with PCOS (**Figure 3**).

In conclusion, these studies strongly suggest the presence of diverse sources of androgen in circulation among women with PCOS. The detection of both classical and 11-oxygenated androgens using liquid chromatography-tandem mass spectrometry (LC-MS/MS) enables the analysis of the comprehensive steroid profile in PCOS women (77). The physiological roles that 11-oxygenated androgens play in the female reproductive system need to be elucidated in future studies.

## 7 The effects of androgen excess in the uterus

A previous study has reported that the mRNA levels of insulin receptor *IRS1/2* and *GLUT1/2* increase in the endometria of patients with PCOS. Chronic DHT treatment affects the expression of genes involved in glucose metabolism and the cell cycle (95). High androgen levels, IR, and a disturbed HPO axis collectively contribute to anovulation and menstrual irregularities. Moreover, the persistent stimulation of estrogen and progesterone in the proliferative phase leads to endometrial overgrowth, impaired endometrial receptivity, and an increased risk of developing endometrial hyperplasia and endometrial cancer in women with PCOS. Obesity can exacerbate these risk factors. In mouse models, hyperandrogenic conditions enhanced the expression of adhesion molecules αvβ3-integrin and lysophosphatidic acid receptor 3 protein, leading to decreased endometrial thickness (96). Endometrial AR expression is overexpressed in patients with PCOS, thus activating TLR4-IRF-7-NFκB signaling and promoting the release of downstream pro-inflammatory cytokines IFNα and IFNγ. Furthermore, the expression levels of ERα and AR increased with the onset of endometrial hyperplasia in women with PCOS (97).

## 8 The effects of androgen excess in other peripheral tissues

Only a small portion of A4 is released into the circulation and then metabolized into other forms of androgens in the periphery, such as the skin, causing dermatological symptoms including hirsutism (increased facial or body hair), acne, and female pattern alopecia (77). The severity of hirsutism can be evaluated using the modified Ferriman–Gallwey (m-FG) score. More recently, Stanczyk et al. identified an indirect DHT formation pathway in the genital skin of patients with PCOS, with the first step being the conversion from A4 to 5α-androstane-3,17-dione (5α-A) via 5α-reductase. Subsequently, 5α-A is metabolized into T by HSD17B5. The study has demonstrated that although serum T and A4 concentrations are significantly higher than 5α-A and DHT, the genital tissue-to-serum ratios indicate opposite results. The ratios in the 5α-A and DHT groups are significantly higher than those in the T and A4 groups. Thus, circulating androgen levels do not completely reflect the local conversion and metabolism of androgen (98).

## 9 Androgen receptor

Classical androgen action is mediated by the nuclear AR. Additionally, androgens exert biological effects via non-genomic signaling involving the MAPK-ERK and AKT pathways. Androgen typically binds to its ARs, whereas DHEA can bind to AR and ER. Distinct differences in AR affinity were observed between different androgen subtypes. DHT has the highest binding affinity of androgens for ARs, followed by T, A4, and DHEA.

Mechanistically, in the absence of a ligand, AR is confined to the cytoplasm and binds to heat shock protein 90 (HSP90) and other co-chaperones. Upon ligand stimulation, HSP90 dissociates from AR, which then localizes to the nucleus and interacts with androgen response elements to initiate the transcription of downstream target genes. Previous studies have demonstrated that longer GGN repeats in the *AR* gene contribute to increased hyperandrogenic states in the Chinese PCOS population, whereas Turkish women with a shorter CAG repeat length had a higher risk for PCOS (99).

AR is widely expressed in female reproductive tissues and its expression is cell- and tissue-specific and fluctuates during the menstrual cycle. Under normal conditions, AR expression is highest in the stroma of the endometrium in the proliferative phase and decreases during the secretory phase until it becomes undetectable. AR expression is present in most stages of follicle development. It increases until the antral stages and then gradually decreases in GCs. Abnormal AR expression is detected in the ovary of patients with PCOS (100). High levels of phosphoglycerate kinase 1, as a binding partner of AR, stabilize ARs and inhibits them from being ubiquitinated through E3 ubiquitin ligase SKP2, thereby promoting AR nuclear translocation and later the expression of ovulation and glucose metabolism-related genes in the GCs of patients with PCOS, thus ultimately leading to anovulation (101). FHL2, a key regulator of follicular development, is also involved in the pathogenesis of anovulation. FHL2 overexpression induced by DHT treatment acts as a co-repressor of AR at the C/EBPβ promoter, thus suppressing C/EBPβ expression, which is a key ovulation-related gene. Furthermore, FHL2 directly inhibits ERK1/2 phosphorylation and downstream gene transcription (102). The inhibition of the ERK1/2 and MEK1/2 pathways is associated with increased androgen biosynthesis, independent of insulin activation (19).

Other studies have reported that AR expression in GCs is significantly decreased among patients with PCOS, which is negatively correlated with LH levels. However, this link disappeared when the number of antral follicles increased to 24 (103). Thus, downregulated ovarian ARs can be compensated by the elevation of androgen levels and increase in extra-ovarian ARs. Another possible explanation is that acute androgen stimulation promotes AR expression in animal models, whereas AR expression is reduced by chronic excess androgen exposure in patients with PCOS (104).

Chronic androgen-driven follicle arrest is caused by abnormal AR degradation, which downregulates RNF6 activity, induces K48-linked polyubiquitination, and then leads to proteasome degradation of AR. Under normal conditions, K63-linked AR polyubiquitination promotes AR transcription activity and the downstream expression of GDF9, a key modulator of follicle growth (105). Consequently, investigating the function of AR and the amount of nuclear import is more important than the abundance of AR expression in patients with PCOS.

Two alternative splice variants (ASVs), Ins-AR and Del-AR, have been identified exclusively in the GCs of patients with PCOS and are closely associated with hyperandrogenism and ovarian dysfunction (106). The former is caused by the insertion of 69 base pairs between exons 2 and 3, whereas the latter lacks exon 3. In addition, patients who carry Ins-AR are positively correlated with high AMH levels; however, although *AR* mRNA is elevated in all groups, no significant difference in the abundance of *AR* mRNA was observed between the AR-SV and wild-type AR (WT-AR) groups (107). This could be explained by alterations in the AR DBD structure of the two isoforms, which then affect the transcription of genes regulating follicular growth and steroidogenesis. CYP17A1 and IGFBP7 expression was upregulated, whereas CYP19A1 expression was suppressed (106). Mechanically, one recent study has revealed that the decreased CYP19A1 expression is driven by the delayed AR nuclear translocation (108). HSP90 exhibits a greater affinity for Ins-AR and Del-AR than WT-AR, making the two AR variants more stable. The Del-AR variant exhibited the greatest binding affinity. Under DHT stimulation, importin α bound more tightly with WT-AR. However, its binding affinity with the Ins-AR and Del-AR isoforms declines dramatically, leading to decreased AR nuclear import. Of note, Ins-AR had the lowest affinity with importin α in response to androgen stimulation. This may explain why patients with the Del-AR variant exhibited a milder PCOS phenotype. Moreover, serine/arginine-rich splicing factors (SRSF), especially SRSF2 and SRSF3, which are regulated by miRNA183 and miRNA124, respectively, participate in the formation of ASVs (107). Xue et al. unveiled more specific molecular mechanisms of how PPT1 induces ovarian HA by disturbing AR transduction in patients with PCOS. In the presence of PPT1, HSP90α is depalmitoylated at the sites of Cys598 and Cys599, and subsequently, damaged AR activation and function. This resulted in the inhibition of downstream *CYP19A1* gene transcription and contributed to the failure of androgen to estrogen conversion. With dipyridamole treatment, the low status of palmitoylated HSP90α was restored, and deleterious effects caused by HA were counteracted (109).

Global or organ-specific AR knockout in DHT-induced mice completely or partially normalized the metabolic and reproductive abnormalities of PCOS, implying that PCOS symptoms are mediated by AR signaling. One study used TC-specific deletion of AR (ThARKO) mice models, only to identify no evident differences in steroid hormone profiles and fertility between the ThARKO group and control group, indicating that these effects are not mediated by AR expressed in TCs. When exposed to excess DHT, ThARKO mice exhibit milder reproductive phenotypes and similar hormone profiles compared with controls. Further studies have indicated that the elevated tissue inhibitor of metalloproteinase 1 (Timp1) is the key player in addressing reproductive dysfunctions. Thus, subfertility is mediated by AR expressed in TCs, although it is not involved in the regulation of sex hormone secretion in PCOS animal models (110). Intriguingly, most traits are still present in GC-specific loss of AR after DHT treatment, whereas anovulation and metabolic dysfunctions, including altered body composition and lipid profiles, are eliminated in neuron-specific AR knockout models (111). More specifically, ovulation is collectively controlled by leptin receptor (LepRb) neurons located in the ARN and the ventral premammillary nuclei in rodents. The study using PNA models with loss of AR in LepRb cells revealed no distinct improvement in metabolic functions and delayed sexual maturation compared with the control group. However, the disrupted estrous cycles were restored to a large degree (112). Muscle-specific AR knockout models displayed similar traits to controls after DHT exposure; however, some aberrant metabolic phenotypes ameliorated in adipose tissue-specific AR knockout mice, but not the reproductive features (113). The deletion of AR in the liver results in restored metabolic functions, such as impaired glucose homeostasis, whereas similar reproductive deficits remained in the control group after DHT exposure (114). Overall, these findings depict the key AR target sites outside of the ovary, including the brain, liver, and adipose tissues, and the crucial roles they play in the development of PCOS. Moreover, the subtype of androgens also matters. AR-knockout protects mice from reproductive and metabolic disorders induced by DHT administration. However, T treatment can still incur reproductive abnormalities. T may indirectly elicit its detrimental reproductive effects by further converting into E2, which can mediate its effects via the ER (115).

Of note, studying animal models mimicking PCOS-like symptoms, which allowed us to elucidate the mechanisms of this condition, had some drawbacks. The postnatal DHT exposure mouse displays typical traits of PCOS without elevated LH secretion. Specifically, despite increased AR expression, chronic androgen stimulation only exerts modest effects on the density of the GnRH spine as well as the number of neurons expressing ERa (116). This may be induced by downregulated kisspeptin activity, thereby decreasing control over GnRH neurons (117). Similarly, one recent study compared several kinds of PCOS animal models at different androgen exposure windows. Gene ontology enrichment analysis revealed that the hypothalamic differentially expressed genes are less enriched in the adult programming group (118). These contradictory results strongly suggest the importance of the timing of androgen exposure and androgen patterns. Although AR expression increases in neuropeptide Y (NPY) neurons, which control eating behaviors, the projections of NPY neurons to GnRH neurons did not change significantly in the PNA model (30). Therefore, different androgen actions may be regulated by different neural circuits.

## 10 Therapeutic strategies

Lifestyle modifications are first-line treatments for PCOS. In recent decades, promising strides have been made in the field of PCOS, and more therapeutic options to restore fertility and ameliorate endocrine and metabolic abnormalities are available. However, most of them are off-label, e.g., insulin-sensitizing agents, some nutritional supplements, and phytochemicals. This Review mainly focuses on alleviating the androgen excess in PCOS patients. Conventional treatments to relieve androgen-related symptoms include oral contraceptive pills and anti-androgen agents.

### 10.1 Oral contraceptive pills

Estrogen reduces the bioactivity of androgen by increasing SHBG contents. One study pointed out that the 6 months of OCP treatments worsened the IR state and hepatic lipid accumulation, which is closely associated with exacerbated inflammation (119, 120). A combined OCP, including estrogen and progestin components, provides greater benefits for HA, but increased thrombotic risk limits its use (121).

### 10.2 Anti-androgen agents

Common anti-androgen medications include flutamide, spironolactone, and finasteride (inhibitor of 5α-reductase). The safety and effects of low-dose flutamide, an androgen receptor antagonist, on alleviating hyperandrogenic symptoms have been well established in clinical practice. Spironolactone, through binding to the AR, has shown strong efficacy in improving hirsutism, acne, and sebum production. However, there are no positive effects on the aspects of BMI, LH, and FSH (122). Notably, contraception is required due to its malformation risk. Abiraterone acetate (AA) administration significantly lowers the 11-oxygenated androgens both in prostate cancer and women with classic 21-hydroxylase deficiency due to its characteristic of inhibiting CYP17A1 activity. However, its role in reducing 11-oxygenated androgens in PCOS needs further investigation (123).

### 10.3 Phytochemicals

In recent years, there has been increased attention on the therapeutic potentials of phytochemicals in the management of PCOS, such as resveratrol, quercetin, proanthocyanidins, berberine, and soy isoflavones. These compounds are mainly derived from flavonoids, polyphenols, and alkaloids. In terms of molecular mechanism, their protection primarily focuses on regulating steroidogenic enzymes, improving IR, and reducing inflammation, apoptosis, and oxidative stress (124). Novel natural compounds like *Tinospora cordifolia* and *Garcinia cambogia* Desr have shown beneficial properties in improving hormonal parameters and restoring ovarian functions (125, 126). Of note, these anti-androgenic and therapeutic effects are mainly validated from the letrozole-induced or DHEA-induced rat models. More randomized controlled trials and further clinical studies investigating efficacy are needed.

### 10.4 Supplements

A study has shown that myo-inositol treatment effectively lowers the LH/FSH ratio and increases the expression of aromatase and FSHR, whereas its isomer D-chiro-Ins (DCI) exhibits the inverse effects on ovarian steroidogenesis (127). Therefore, it has been reported that when given DCI and myo-inositol at a ratio of 1:40, it can significantly mitigate the abnormalities of PCOS and improve insulin sensitivity (128). Evidence has supported the close relationship between vitamin D (VD) and female fertility, regulating IR, inflammatory response, and oxidative stress. It has been reported that there is a higher prevalence of VD deficiency in PCOS patients with HA. VD supplementation in women with PCOS is helpful in improving PCOS symptoms and related indices without adverse effects (129).

### 10.5 Nanotechnologies

Selenium nanoparticles (SeNPs) are promising tools with lower toxicity and enhanced bioavailability to target multiple diseases for their anti-inflammatory and anti-antioxidant properties (130). One study demonstrated that SeNPs reduce androgen production by downregulating the expression of key steroidogenic enzymes, including STAR, CYP11A1, CYP17A1, and HSD17B3, while increasing the expression of CYP19α1. Additionally, the block of AR expression was found (131). Fourteen days of low-dose SeNP supplementation normalizes the aberrant metabolic features of PCOS and inhibits the expression of inflammatory factors, including IL-6, TNF-α, and IL-1, via repairing the antioxidant KEAP1/NRF2 cascades (132). The combination with metformin largely alleviates IR and metabolic abnormalities by upregulating PI3K-AKT signaling (133).

### 10.6 Target circadian rhythm

In recent years, promising therapies modifying circadian rhythm to alleviate HA have been proposed. Long-term exposure to light would result in increased FSH and estradiol levels. Interestingly, prolonged darkness would lead to HA and IR in PCOS rat models. Downregulated melatonin receptor 1A disturbs AR expression and downstream CYP19A1 transcription (134). Another study revealed that the dysregulation of some core clock gene expression is closely associated with HA. CYP17A1 has been identified as the direct target of the brain and muscle ARNT-like 1 (*BMAL1*) gene in peripheral blood mononuclear cells (PBMCs), and low expression of BMAL1 increased the activity of 5α-reductase, catalyzing more T into DHT (135). In the liver, BMAL1 inhibits GLUT4 expression, whereas period (PER1/2) interferes with AR and the production of hepatic IGFBP4 and SHBG, ultimately causing IR and androgen excess (136). Moreover, androgen directly disrupts the hepatic timing system, ultimately impairing liver metabolism through methylation modification of H3K27me3 (137). Additionally, BMAL1 is considered an activator of luteinizing hormone receptor (LHCGR) transcription, which promotes LH surge and ovulation (138). Clinical trials have shown that the administration of melatonin at 5 mg for 12 weeks significantly lowers the levels of T and biomarkers of inflammation and alleviates hirsutism in women with PCOS (139).

### 10.7 Target neuroendocrine dysfunction

New therapies targeting the abnormal GnRH neuron circuits have been developed. Neurokinin 3 receptor (NK3R) antagonist (MLE4901) mitigates GnRH pulsatility, androgen excess, and metabolic abnormalities in a DHT-induced PCOS mouse model, with no effect on reproductive traits (140). Notably, antagonizing the local ovarian NK3R systems also alleviates the PCOS-like symptoms. In terms of mechanism, treatment with NK3R antagonist restored mitochondrial function and decreased oxidative stress in KGN cells (141). In addition, as mentioned before, NPY is found to participate in the modulation of reproduction and metabolism in rats. Hypothalamic NPY enhances GnRH-stimulated LH secretion and inhibits kisspeptin. It has been shown that NPY Y5 receptor antagonists significantly suppress weight gain and food intake (142). Further research and clinical trials are required before this neuropeptide can be used to treat the metabolic and reproductive dysfunctions observed in PCOS.

### 10.8 Mesenchymal stem cell therapy

Recently, mesenchymal stem cell (MSC) therapies have been seen as promising and safe tools to reverse some PCOS traits, including mitigating inflammation and restoring fertility. After the injection of human bone marrow MSCs into the ovary, androgen-synthesizing gene expression, including *CYP17A1* and *DENND1A*, was significantly downregulated, while *FSHR* and *CYP19A1* gene expression was upregulated. These effects were partly mediated by secretome components IL-10 and BMP-2 (143, 144). One study further uncovered several key protein components from hMSCs regulating androgen production (145).

## 11 Conclusion and future directions

HA is a key feature of PCOS. The utilization of selective or complete AR knockout animal models has revealed that HA induces PCOS traits via AR and highlights the extra ovarian target sites of AR action, including the brain, adipose tissue, and liver, of which the brain is the core site.

The current therapeutic approaches aimed at alleviating hyperandrogenic manifestations may not be applicable to patients with phenotype D, based on the Rotterdam classification. Conventionally, biochemical androgen measurements primarily focus on total T or T levels and the free androgen index. The application of LC-MS/MS enables the simultaneous measurement of several kinds of steroid hormones in women with PCOS. Recently, A4 has been identified as a sensitive marker for identifying women with hyperandrogenic PCOS. Clinical hyperandrogenism is defined as the presence of hirsutism recorded as an m-FG score of ≥8, which can become unreliable when applied to different ethnicities. Therefore, these traditional perspectives have been challenged. Apart from the previous findings that ovarian androgen is a dominant source of androgen excess in women with PCOS, adrenal-derived androgen, particularly 11-oxygenated androgen, also contributes to the circulating androgen pools.

HA in PCOS is characterized by an overall elevation of serum androgen levels via the involvement of classic, 11-oxygenated, and backdoor pathways (146). Therefore, future research should aim to identify more precise detection methods covering the entire androgen profile and quantity of androgen, as well as variations in the menstrual cycle, circadian rhythms, age, BMI, and samples from different collection sites (saliva, urine, and blood), which may all potentially influence the measurement of androgen levels (89). Moreover, serum androgen levels are not completely equivalent to the local conversion of androgen. Different bioactivities and binding affinities of androgens to AR are also important.

In conclusion, the current understanding of the regulatory role of androgens in the development of PCOS remains limited; therefore, continuous efforts to determine the exact mechanism of this condition are required.

## Author contributions

KW: Conceptualization, Investigation, Methodology, Writing – original draft, Writing – review & editing. YL: Supervision, Writing – review & editing. YC: Methodology, Writing – review & editing.

## References

[1] Walters KA Gilchrist RB Ledger WL Teede HJ Handelsman DJ Campbell RE. New perspectives on the pathogenesis of PCOS: neuroendocrine origins. Trends Endocrinol metabolism: TEM (2018) 29(12):841–52. doi: 10.1016/j.tem.2018.08.005 30195991

[2] Unfer V Dinicola S. Russo M. A PCOS paradox: does inositol therapy find a rationale in all the different phenotypes? Int J Mol Sci (2023) 24(7):6213. doi: 10.3390/ijms24076213 37047186 PMC10094056

[3] Siddiqui S Mateen S Ahmad R Moin S. A brief insight into the etiology, genetics, and immunology of polycystic ovarian syndrome (PCOS). J assisted Reprod Genet (2022) 39(11):2439–73. doi: 10.1007/s10815-022-02625-7 PMC9723082 36190593

[4] Kim JJ Kim D Yim JY Kang JH Han KH Kim SM. Polycystic ovary syndrome with hyperandrogenism as a risk factor for non-obese non-alcoholic fatty liver disease. Alimentary Pharmacol Ther (2017) 45(11):1403–12. doi: 10.1111/apt.14058 28370150

[5] Persson S Elenis E Turkmen S Kramer MS Yong EL Poromaa IS. Higher risk of type 2 diabetes in women with hyperandrogenic polycystic ovary syndrome. Fertil steril Sep (2021) 116(3):862–71. doi: 10.1016/j.fertnstert.2021.04.018 34053678

[6] Stone T Yanes Cardozo LL Oluwatade TN Leone CA Burgos M Okifo F. Testosterone-associated blood pressure dysregulation in women with androgen excess polycystic ovary syndrome. Am J Physiol Heart Circulatory Physiol (2023) 325(2):H232–h243. doi: 10.1152/ajpheart.00164.2023 PMC10393337 37327000

[7] Risal S Pei Y Lu H Manti M Fornes R Pui HP. Prenatal androgen exposure and transgenerational susceptibility to polycystic ovary syndrome. Nat Med (2019) 25(12):1894–904. doi: 10.1038/s41591-019-0666-1 31792459

[8] Krentowska A Kowalska I. Metabolic syndrome and its components in different phenotypes of polycystic ovary syndrome. Diabetes/metabolism Res Rev (2022) 38(1):e3464. doi: 10.1002/dmrr.3464 33988288

[9] Gleicher N Darmon S Patrizio P Barad DH. Reconsidering the polycystic ovary syndrome (PCOS). Biomedicines (2022) 10(7):1505. doi: 10.3390/biomedicines10071505 35884809 PMC9313207

[10] Myers SH Russo M Dinicola S Forte G Unfer V. Questioning PCOS phenotypes for reclassification and tailored therapy. Trends Endocrinol metabolism: TEM (2023) 34:694–703. doi: 10.1016/j.tem.2023.08.005 37661546

[11] Zhao H Zhang J Cheng X Nie X He B. Insulin resistance in polycystic ovary syndrome across various tissues: an updated review of pathogenesis, evaluation, and treatment. J Ovarian Res (2023) 16(1):9. doi: 10.1186/s13048-022-01091-0 36631836 PMC9832677

[12] Li H Chen Y Yan LY Qiao J. Increased expression of P450scc and CYP17 in development of endogenous hyperandrogenism in a rat model of PCOS. Endocrine (2013) 43(1):184–90. doi: 10.1007/s12020-012-9739-3 22798247

[13] Tosi F Negri C Brun E Castello R Faccini G Bonora E. Insulin enhances ACTH-stimulated androgen and glucocorticoid metabolism in hyperandrogenic women. Eur J endocrinol (2011) 164(2):197–203. doi: 10.1530/eje-10-0782 21059865

[14] Wu C Wei K Jiang Z. 5α-reductase activity in women with polycystic ovary syndrome: a systematic review and meta-analysis. Reprod Biol Endocrinol RB&E (2017) 15(1):21. doi: 10.1186/s12958-017-0242-9 28347315 PMC5369013

[15] Liu Y Wang Y Yao D Chen X Zhang F Feng Y. Diane-35 and metformin induce autophagy and apoptosis in polycystic ovary syndrome women with early-stage endometrial carcinoma. Genes (2022) 13(1):131. doi: 10.3390/genes13010131 35052471 PMC8775133

[16] Dai S Zhang H Yang F Shang W Zeng S. Effects of IGF-1 on the three-dimensional culture of ovarian preantral follicles and superovulation rates in mice. Biology (2022) 11(6):833. doi: 10.3390/biology11060833 35741354 PMC9219699

[17] Munir I Yen HW Geller DH Torbati D Bierden RM Weitsman SR. Insulin augmentation of 17alpha-hydroxylase activity is mediated by phosphatidyl inositol 3-kinase but not extracellular signal-regulated kinase-1/2 in human ovarian theca cells. Endocrinology (2004) 145(1):175–83. doi: 10.1210/en.2003-0329 14512432

[18] Book CB Dunaif A. Selective insulin resistance in the polycystic ovary syndrome. J Clin Endocrinol Metab (1999) 84(9):3110–6. doi: 10.1210/jcem.84.9.6010 10487672

[19] Diamanti-Kandarakis E Argyrakopoulou G Economou F Kandaraki E Koutsilieris M. Defects in insulin signaling pathways in ovarian steroidogenesis and other tissues in polycystic ovary syndrome (PCOS). J Steroid Biochem Mol Biol (2008) 109(3-5):242–6. doi: 10.1016/j.jsbmb.2008.03.014 18440223

[20] Liao B Qi X Yun C Qiao J Pang Y. Effects of androgen excess-related metabolic disturbances on granulosa cell function and follicular development. Front endocrinol (2022) 13:815968. doi: 10.3389/fendo.2022.815968 PMC8883052 35237237

[21] di Clemente N Racine C Pierre A Taieb J. Anti-müllerian hormone in female reproduction. Endocr Rev (2021) 42(6):753–82. doi: 10.1210/endrev/bnab012 33851994

[22] Dilaver N Pellatt L Jameson E Ogunjimi M Bano G Homburg R. The regulation and signalling of anti-Müllerian hormone in human granulosa cells: relevance to polycystic ovary syndrome. Hum Reprod (Oxford England) (2019) 34(12):2467–79. doi: 10.1093/humrep/dez214 31735954

[23] Li Y Zhai Y Li L Lu Y Su S Liu Y. Divergent associations between serum androgens and ovarian reserve markers revealed in patients with polycystic ovary syndrome. Front endocrinol (2022) 13:881740. doi: 10.3389/fendo.2022.881740 PMC9218193 35757414

[24] Sivanandy MS Ha SK. The role of serum anti-mullerian hormone measurement in the diagnosis of polycystic ovary syndrome. Diagnostics (Basel Switzerland) (2023) 13(5):907. doi: 10.3390/diagnostics13050907 36900051 PMC10000702

[25] Barbotin AL Mimouni NEH Kuchcinski G Lopes R Viard R Rasika S. Hypothalamic neuroglial plasticity is regulated by anti-Müllerian hormone and disrupted in polycystic ovary syndrome. EBioMedicine Apr (2023) 90:104535. doi: 10.1016/j.ebiom.2023.104535 PMC10070524 37001236

[26] Moore AM. Impaired steroid hormone feedback in polycystic ovary syndrome: Evidence from preclinical models for abnormalities within central circuits controlling fertility. Clin endocrinol Aug (2022) 97(2):199–207. doi: 10.1111/cen.14711 PMC11289760 35349177

[27] Okada H Kanasaki H Tumurbaatar T Tumurgan Z Oride A Kyo S. Hyperandrogenism induces proportional changes in the expression of Kiss-1, Tac2, and DynA in hypothalamic KNDy neurons. Reprod Biol Endocrinol RB&E (2022) 20(1):91. doi: 10.1186/s12958-022-00963-w 35729637 PMC9210811

[28] Garg A Patel B Abbara A Dhillo WS. Treatments targeting neuroendocrine dysfunction in polycystic ovary syndrome (PCOS). Clin endocrinol (2022) 97(2):156–64. doi: 10.1111/cen.14704 35262967

[29] Silva MSB Desroziers E Hessler S Prescott M Coyle C Herbison AE. Activation of arcuate nucleus GABA neurons promotes luteinizing hormone secretion and reproductive dysfunction: Implications for polycystic ovary syndrome. EBioMedicine (2019) 44:582–96. doi: 10.1016/j.ebiom.2019.05.065 PMC6606966 31178425

[30] Marshall CJ Prescott M Campbell RE. Investigating the NPY/agRP/GABA to gnRH neuron circuit in prenatally androgenized PCOS-like mice. J Endocr Society (2020) 4(11):bvaa129. doi: 10.1210/jendso/bvaa129 PMC7566551 33094210

[31] Venkatesh VS Grossmann M Zajac JD Davey RA. The role of the androgen receptor in the pathogenesis of obesity and its utility as a target for obesity treatments. Obes Rev an Off J Int Assoc Study Obes (2022) 23(6):e13429. doi: 10.1111/obr.13429 PMC9286619 35083843

[32] Ruth KS Day FR Tyrrell J Thompson DJ Wood AR Mahajan A. Using human genetics to understand the disease impacts of testosterone in men and women. Nat Med (2020) 26(2):252–8. doi: 10.1038/s41591-020-0751-5 PMC7025895 32042192

[33] Escobar-Morreale HF Martínez-García MÁ Insenser M Cañellas N Correig X Luque-Ramírez M. Serum metabolomics profiling by proton nuclear magnetic resonance spectroscopy reveals sexual dimorphism and masculinization of intermediate metabolism in women with polycystic ovary syndrome (PCOS). Biol sex differences (2023) 14(1):21. doi: 10.1186/s13293-023-00507-w PMC10114375 37076926

[34] Schiffer L Kempegowda P Arlt W O’Reilly MW. MECHANISMS IN ENDOCRINOLOGY: The sexually dimorphic role of androgens in human metabolic disease. Eur J endocrinol (2017) 177(3):R125–r143. doi: 10.1530/eje-17-0124 28566439 PMC5510573

[35] Escobar-Morreale HF Alvarez-Blasco F Botella-Carretero JI Luque-Ramírez M. The striking similarities in the metabolic associations of female androgen excess and male androgen deficiency. Hum Reprod (Oxford England) (2014) 29(10):2083–91. doi: 10.1093/humrep/deu198 25104855

[36] Castillo-Higuera T Alarcón-Granados MC Marin-Suarez J Moreno-Ortiz H Esteban-Pérez CI Ferrebuz-Cardozo AJ. A comprehensive overview of common polymorphic variants in genes related to polycystic ovary syndrome. Reprod Sci (Thousand Oaks Calif) (2021) 28(9):2399–412. doi: 10.1007/s43032-020-00375-4 33174186

[37] Heidarzadehpilehrood R Pirhoushiaran M Abdollahzadeh R Binti Osman M Sakinah M Nordin N. A review on CYP11A1, CYP17A1, and CYP19A1 polymorphism studies: candidate susceptibility genes for polycystic ovary syndrome (PCOS) and infertility. Genes (2022) 13:302. doi: 10.3390/genes13020302 35205347 PMC8871850

[38] Pusalkar M Meherji P Gokral J Chinnaraj S Maitra A. CYP11A1 and CYP17 promoter polymorphisms associate with hyperandrogenemia in polycystic ovary syndrome. Fertil steril (2009) 92(2):653–9. doi: 10.1016/j.fertnstert.2008.07.016 18725155

[39] Kevenaar ME Laven JS Fong SL Uitterlinden AG de Jong FH Themmen AP. A functional anti-mullerian hormone gene polymorphism is associated with follicle number and androgen levels in polycystic ovary syndrome patients. J Clin Endocrinol Metab (2008) 93(4):1310–6. doi: 10.1210/jc.2007-2205 18230658

[40] Moolhuijsen LME Louwers YV McLuskey A Broer L Uitterlinden AG Verdiesen RMG. Association between an AMH promoter polymorphism and serum AMH levels in PCOS patients. Hum Reprod (Oxford England) (2022) 37(7):1544–56. doi: 10.1093/humrep/deac082 PMC9247424 35451015

[41] Li Y Fang L Yan Y Wang Z Wu Z Jia Q. Association between human SHBG gene polymorphisms and risk of PCOS: a meta-analysis. Reprod biomed online (2021) 42(1):227–36. doi: 10.1016/j.rbmo.2020.10.003 33168491

[42] Alemany M. The roles of androgens in humans: biology, metabolic regulation and health. Int J Mol Sci (2022) 23(19):11952. doi: 10.3390/ijms231911952 36233256 PMC9569951

[43] Stener-Victorin E Holm G Labrie F Nilsson L Janson PO Ohlsson C. Are there any sensitive and specific sex steroid markers for polycystic ovary syndrome? J Clin Endocrinol Metab (2010) 95(2):810–9. doi: 10.1210/jc.2009-1908 20016048

[44] Ge J Yang N Zhang X Li M Zhang W He J. Steroid hormone profiling in hyperandrogenism and non-hyperandrogenism women with polycystic ovary syndrome. Reprod Sci (Thousand Oaks Calif) (2022) 29(12):3449–58. doi: 10.1007/s43032-022-00985-0 35835901

[45] Marti N Galván JA Pandey AV Trippel M Tapia C Müller M. Genes and proteins of the alternative steroid backdoor pathway for dihydrotestosterone synthesis are expressed in the human ovary and seem enhanced in the polycystic ovary syndrome. Mol Cell endocrinol (2017) 441:116–23. doi: 10.1016/j.mce.2016.07.029 27471004

[46] Sanchez-Garrido MA Tena-Sempere M. Metabolic dysfunction in polycystic ovary syndrome: Pathogenic role of androgen excess and potential therapeutic strategies. Mol Metab (2020) 35:100937. doi: 10.1016/j.molmet.2020.01.001 32244180 PMC7115104

[47] McAllister JM Modi B Miller BA Biegler J Bruggeman R Legro RS. Overexpression of a DENND1A isoform produces a polycystic ovary syndrome theca phenotype. Proc Natl Acad Sci USA (2014) 111(15):E1519–27. doi: 10.1073/pnas.1400574111 PMC3992676 24706793

[48] McFee RM Romereim SM Snider AP Summers AF Pohlmeier WE Kurz SG. A high-androgen microenvironment inhibits granulosa cell proliferation and alters cell identity. Mol Cell endocrinol (2021) 531:111288. doi: 10.1016/j.mce.2021.111288 33905753 PMC13104108

[49] Zeng X Zhong Q Li M Liu Y Long S Xie Y. Androgen increases klotho expression via the androgen receptor-mediated pathway to induce GCs apoptosis. J Ovarian Res (2023) 16(1):10. doi: 10.1186/s13048-022-01087-w 36641458 PMC9840339

[50] Candelaria NR Padmanabhan A Stossi F Ljungberg MC Shelly KE Pew BK. VCAM1 is induced in ovarian theca and stromal cells in a mouse model of androgen excess. Endocrinology (2019) 160(6):1377–93. doi: 10.1210/en.2018-00731 PMC6507908 30951142

[51] Wang J Yin T Liu S. Dysregulation of immune response in PCOS organ system. Front Immunol (2023) 14:1169232. doi: 10.3389/fimmu.2023.1169232 37215125 PMC10196194

[52] Fox CW Zhang L Sohni A Doblado M Wilkinson MF Chang RJ. Inflammatory stimuli trigger increased androgen production and shifts in gene expression in theca-interstitial cells. Endocrinology (2019) 160(12):2946–58. doi: 10.1210/en.2019-00588 PMC6855291 31599939

[53] Li Y Zheng Q Sun D Cui X Chen S Bulbul A. Dehydroepiandrosterone stimulates inflammation and impairs ovarian functions of polycystic ovary syndrome. J Cell Physiol (2019) 234(5):7435–47. doi: 10.1002/jcp.27501 30580448

[54] Li X Pishdari B Cui P Hu M Yang HP Guo YR. Regulation of androgen receptor expression alters AMPK phosphorylation in the endometrium: *in vivo* and *in vitro* studies in women with polycystic ovary syndrome. Int J Biol Sci (2015) 11(12):1376–89. doi: 10.7150/ijbs.13109 PMC4671995 26681917

[55] González F Sia CL Bearson DM Blair HE. Hyperandrogenism induces a proinflammatory TNFα response to glucose ingestion in a receptor-dependent fashion. J Clin Endocrinol Metab (2014) 99(5):E848–54. doi: 10.1210/jc.2013-4109 PMC4010708 24512496

[56] Wagner IV Savchuk I Sahlin L Kulle A Klöting N Dietrich A. *De novo* and depot-specific androgen production in human adipose tissue: A source of hyperandrogenism in women with obesity. Obes facts (2022) 15(2):281–91. doi: 10.1159/000521571 PMC9021649 34983051

[57] Wang L Li S Zhao A Tao T Mao X Zhang P. The expression of sex steroid synthesis and inactivation enzymes in subcutaneous adipose tissue of PCOS patients. J Steroid Biochem Mol Biol (2012) 132(1-2):120–6. doi: 10.1016/j.jsbmb.2012.02.003 22381227

[58] O'Reilly M Gathercole L Capper F Arlt W Tomlinson J. Effect of insulin on AKR1C3 expression in female adipose tissue: *in-vivo* and *in-vitro* study of adipose androgen generation in polycystic ovary syndrome. Lancet (London England) (2015) 385 Suppl 1:S16. doi: 10.1016/s0140-6736(15)60331-2 26312838

[59] Ostinelli G Laforest S Denham SG Gauthier MF Drolet-Labelle V Scott E. Increased adipose tissue indices of androgen catabolism and aromatization in women with metabolic dysfunction. J Clin Endocrinol Metab (2022) 107(8):e3330–42. doi: 10.1210/clinem/dgac261 PMC9282357 35511873

[60] Shabbir S Khurram E Moorthi VS Eissa YTH Kamal MA Butler AE. The interplay between androgens and the immune response in polycystic ovary syndrome. J Trans Med (2023) 21(1):259. doi: 10.1186/s12967-023-04116-4 PMC10105935 37062827

[61] de Medeiros SF Rodgers RJ Norman RJ. Adipocyte and steroidogenic cell cross-talk in polycystic ovary syndrome. Hum Reprod update (2021) 27(4):771–96. doi: 10.1093/humupd/dmab004 33764457

[62] Dumesic DA Phan JD Leung KL Grogan TR Ding X Li X. Adipose insulin resistance in normal-weight women with polycystic ovary syndrome. J Clin Endocrinol Metab (2019) 104(6):2171–83. doi: 10.1210/jc.2018-02086 PMC6482023 30649347

[63] Divoux A Erdos E Whytock K Osborne TF Smith SR. Transcriptional and DNA methylation signatures of subcutaneous adipose tissue and adipose-derived stem cells in PCOS women. Cells (2022) 11(5):848. doi: 10.3390/cells11050848 35269469 PMC8909136

[64] Xu N Kwon S Abbott DH Geller DH Dumesic DA Azziz R. Epigenetic mechanism underlying the development of polycystic ovary syndrome (PCOS)-like phenotypes in prenatally androgenized rhesus monkeys. PloS One (2011) 6(11):e27286. doi: 10.1371/journal.pone.0027286 22076147 PMC3208630

[65] Lemaitre M Christin-Maitre S Kerlan V. Polycystic ovary syndrome and adipose tissue. Annales d'endocrinologie (2023) 84(2):308–15. doi: 10.1016/j.ando.2022.11.004 36623807

[66] Naamneh Elzenaty R du Toit T Flück CE. Basics of androgen synthesis and action. Best Pract Res Clin Endocrinol Metab (2022) 36(4):101665. doi: 10.1016/j.beem.2022.101665 35595638

[67] Ye W Xie T Song Y Zhou L. The role of androgen and its related signals in PCOS. J Cell Mol Med (2021) 25(4):1825–37. doi: 10.1111/jcmm.16205 PMC7882969 33369146

[68] Goodarzi MO Antoine HJ Azziz R. Genes for enzymes regulating dehydroepiandrosterone sulfonation are associated with levels of dehydroepiandrosterone sulfate in polycystic ovary syndrome. J Clin Endocrinol Metab (2007) 92(7):2659–64. doi: 10.1210/jc.2006-2600 17426092

[69] Wang J Wu D Guo H Li M. Hyperandrogenemia and insulin resistance: The chief culprit of polycystic ovary syndrome. Life Sci (2019) 236:116940. doi: 10.1016/j.lfs.2019.116940 31604107

[70] Liang J Zhang B Hu Y Na Z Li D. Effects of steroid hormones on lipid metabolism in sexual dimorphism: A Mendelian randomization study. Front endocrinol (2022) 13:1119154. doi: 10.3389/fendo.2022.1119154 PMC9886494 36726474

[71] Goodarzi MO Carmina E Azziz R. DHEA, DHEAS and PCOS. J Steroid Biochem Mol Biol (2015) 145:213–25. doi: 10.1016/j.jsbmb.2014.06.003 25008465

[72] Vryonidou A Papatheodorou A Tavridou A Terzi T Loi V Vatalas IA. Association of hyperandrogenemic and metabolic phenotype with carotid intima-media thickness in young women with polycystic ovary syndrome. J Clin Endocrinol Metab (2005) 90(5):2740–6. doi: 10.1210/jc.2004-2363 15741256

[73] Chen F Chen M Zhang W Yin H Chen G Huang Q. Comparison of the efficacy of different androgens measured by LC-MS/MS in representing hyperandrogenemia and an evaluation of adrenal-origin androgens with a dexamethasone suppression test in patients with PCOS. J Ovarian Res (2021) 14(1):32. doi: 10.1186/s13048-021-00781-5 33583431 PMC7883427

[74] Turcu AF Rege J Auchus RJ Rainey WE. 11-Oxygenated androgens in health and disease. Nat Rev Endocrinol (2020) 16(5):284–96. doi: 10.1038/s41574-020-0336-x PMC7881526 32203405

[75] Turcu AF Nanba AT Auchus RJ. The rise, fall, and resurrection of 11-oxygenated androgens in human physiology and disease. Hormone Res paediatrics (2018) 89(5):284–91. doi: 10.1159/000486036 PMC6031471 29742491

[76] Handelsman DJ Cooper ER Heather AK. Bioactivity of 11 keto and hydroxy androgens in yeast and mammalian host cells. J Steroid Biochem Mol Biol (2022) 218:106049. doi: 10.1016/j.jsbmb.2021.106049 34990809

[77] Storbeck KH O'Reilly MW. The clinical and biochemical significance of 11-oxygenated androgens in human health and disease. Eur J endocrinol (2023) 188(4):R98–r109. doi: 10.1093/ejendo/lvad047 37041725

[78] Imamichi Y Yuhki KI Orisaka M Kitano T Mukai K Ushikubi F. 11-ketotestosterone is a major androgen produced in human gonads. J Clin Endocrinol Metab (2016) 101(10):3582–91. doi: 10.1210/jc.2016-2311 27428878

[79] Auer MK Hawley JM Lottspeich C Bidlingmaier M Sappl A Nowotny HF. 11-Oxygenated androgens are not secreted by the human ovary: in-vivo data from four different cases of hyperandrogenism. Eur J endocrinol (2022) 187(6):K47–k53. doi: 10.1530/eje-22-0518 36239921 PMC9716487

[80] Nanba AT Rege J Ren J Auchus RJ Rainey WE Turcu AF. 11-oxygenated C19 steroids do not decline with age in women. J Clin Endocrinol Metab (2019) 104(7):2615–22. doi: 10.1210/jc.2018-02527 PMC6525564 30753518

[81] Moulana M. Androgen-induced cardiovascular risk in polycystic ovary syndrome: the role of T lymphocytes. Life (Basel Switzerland) (2023) 13(4):1010. doi: 10.3390/life13041010 37109539 PMC10145997

[82] Hirschberg AL. Approach to investigation of hyperandrogenism in a postmenopausal woman. J Clin Endocrinol Metab (2023) 108(5):1243–53. doi: 10.1210/clinem/dgac673 PMC10099172 36409990

[83] Markopoulos MC Rizos D Valsamakis G Deligeoroglou E Grigoriou O Chrousos GP. Hyperandrogenism in women with polycystic ovary syndrome persists after menopause. J Clin Endocrinol Metab (2011) 96(3):623–31. doi: 10.1210/jc.2010-0130 21177795

[84] Taylor AE Ware MA Breslow E Pyle L Severn C Nadeau KJ. 11-oxyandrogens in adolescents with polycystic ovary syndrome. J Endocr Society (2022) 6(7):bvac037. doi: 10.1210/jendso/bvac037 PMC9123281 35611324

[85] Torchen LC Sisk R Legro RS Turcu AF Auchus RJ Dunaif A. 11-oxygenated C19 steroids do not distinguish the hyperandrogenic phenotype of PCOS daughters from girls with obesity. J Clin Endocrinol Metab (2020) 105(11):e3903–9. doi: 10.1210/clinem/dgaa532 PMC7500474 32797203

[86] Rege J Turcu AF Kasa-Vubu JZ Lerario AM Auchus GC Auchus RJ. 11-ketotestosterone is the dominant circulating bioactive androgen during normal and premature adrenarche. J Clin Endocrinol Metab (2018) 103(12):4589–98. doi: 10.1210/jc.2018-00736 PMC6226603 30137510

[87] O'Reilly MW Kempegowda P Jenkinson C Taylor AE Quanson JL Storbeck KH. 11-oxygenated C19 steroids are the predominant androgens in polycystic ovary syndrome. J Clin Endocrinol Metab (2017) 102(3):840–8. doi: 10.1210/jc.2016-3285 PMC5460696 27901631

[88] Yoshida T Matsuzaki T Miyado M Saito K Iwasa T Matsubara Y. 11-oxygenated C19 steroids as circulating androgens in women with polycystic ovary syndrome. Endocr J (2018) 65(10):979–90. doi: 10.1507/endocrj.EJ18-0212 30012903

[89] Schiffer L Kempegowda P Sitch AJ Adaway JE Shaheen F Ebbehoj A. Classic and 11-oxygenated androgens in serum and saliva across adulthood: a cross-sectional study analyzing the impact of age, body mass index, and diurnal and menstrual cycle variation. Eur J endocrinol (2023) 188(1):lvac017. doi: 10.1093/ejendo/lvac017 36651154

[90] Dumesic DA Turcu AF Liu H Grogan TR Abbott DH Lu G. Interplay of cortisol, testosterone, and abdominal fat mass in normal-weight women with polycystic ovary syndrome. J Endocr Society (2023) 7(8):bvad079. doi: 10.1210/jendso/bvad079 PMC10315644 37404244

[91] Walzer D Turcu AF Jha S Abel BS Auchus RJ Merke DP. Excess 11-oxygenated androgens in women with severe insulin resistance are mediated by adrenal insulin receptor signaling. J Clin Endocrinol Metab (2022) 107(9):2626–35. doi: 10.1210/clinem/dgac365 PMC9387696 35696182

[92] Kinyua AW Doan KV Yang DJ Huynh MKQ Choi YH Shin DM. Insulin regulates adrenal steroidogenesis by stabilizing SF-1 activity. Sci Rep (2018) 8(1):5025. doi: 10.1038/s41598-018-23298-2 29567944 PMC5864882

[93] Paulukinas RD Mesaros CA Penning TM. Conversion of classical and 11-oxygenated androgens by insulin-induced AKR1C3 in a model of human PCOS adipocytes. Endocrinology (2022) 163(7):bqac068. doi: 10.1210/endocr/bqac068 35560164 PMC9162389

[94] Paulukinas RD Penning TM. Insulin-induced AKR1C3 induces fatty acid synthase in a model of human PCOS adipocytes. Endocrinology (2023) 164(5):bqad033. doi: 10.1210/endocr/bqad033 36799021 PMC10282923

[95] Lee MH Yoon JA Kim HR Kim YS Lyu SW Lee BS. Hyperandrogenic milieu dysregulates the expression of insulin signaling factors and glucose transporters in the endometrium of patients with polycystic ovary syndrome. Reprod Sci (Thousand Oaks Calif) (2020) 27(8):1637–47. doi: 10.1007/s43032-020-00194-7 32430710

[96] Wang Z Nie K Su H Tang Y Wang H Xu X. Berberine improves ovulation and endometrial receptivity in polycystic ovary syndrome. Phytomed Int J phytotherapy phytopharmacol (2021) 91:153654. doi: 10.1016/j.phymed.2021.153654 34333328

[97] Hu M Zhang Y Li X Cui P Sferruzzi-Perri AN Brännström M. TLR4-associated IRF-7 and NFκB signaling act as a molecular link between androgen and metformin activities and cytokine synthesis in the PCOS endometrium. J Clin Endocrinol Metab (2021) 106(4):1022–40. doi: 10.1210/clinem/dgaa951 33382900

[98] Stanczyk FZ Mandelbaum R Baker M Ma L Sriprasert I Dancz CE. Quantitation of 5α-androstanedione in normal women and women with PCOS. J Steroid Biochem Mol Biol (2023) 231:106289. doi: 10.1016/j.jsbmb.2023.106289 36972792

[99] Yuan C Gao C Qian Y Liu Y Jiang SW Cui Y. Polymorphism of CAG and GGN repeats of androgen receptor gene in women with polycystic ovary syndrome. Reprod biomed online (2015) 31(6):790–8. doi: 10.1016/j.rbmo.2015.09.007 26511871

[100] Walters KA Rodriguez Paris V Aflatounian A Handelsman DJ. Androgens and ovarian function: translation from basic discovery research to clinical impact. J endocrinol (2019) 242(2):R23–r50. doi: 10.1530/joe-19-0096 31125975

[101] Liu X Sun C Zou K Li C Chen X Gu H. Novel PGK1 determines SKP2-dependent AR stability and reprograms granular cell glucose metabolism facilitating ovulation dysfunction. EBioMedicine (2020) 61:103058. doi: 10.1016/j.ebiom.2020.103058 33096483 PMC7581881

[102] Zhou R Li S Liu J Wu H Yao G Sun Y. Up-regulated FHL2 inhibits ovulation through interacting with androgen receptor and ERK1/2 in polycystic ovary syndrome. EBioMedicine (2020) 52:102635. doi: 10.1016/j.ebiom.2020.102635 32028069 PMC6997507

[103] Gao XY Liu Y Lv Y Huang T Lu G Liu HB. Role of androgen receptor for reconsidering the "True" Polycystic ovarian morphology in PCOS. Sci Rep (2020) 10(1):8993. doi: 10.1038/s41598-020-65890-5 32488141 PMC7265442

[104] Abdul Hamid F Abu MA Abdul Karim AK Ahmad MF Abd Aziz NH Mohd Kamal DA. Sex steroid receptors in polycystic ovary syndrome and endometriosis: insights from laboratory studies to clinical trials. Biomedicines (2022) 10(7):1705. doi: 10.3390/biomedicines10071705 35885010 PMC9312843

[105] Lim JJ Lima PDA Salehi R Lee DR Tsang BK. Regulation of androgen receptor signaling by ubiquitination during folliculogenesis and its possible dysregulation in polycystic ovarian syndrome. Sci Rep (2017) 7(1):10272. doi: 10.1038/s41598-017-09880-0 28860512 PMC5578986

[106] Wang F Pan J Liu Y Meng Q Lv P Qu F. Alternative splicing of the androgen receptor in polycystic ovary syndrome. Proc Natl Acad Sci USA (2015) 112(15):4743–8. doi: 10.1073/pnas.1418216112 PMC4403157 25825716

[107] Luo J Ye H Hao L Sun Y Li R Li Y. SRSFs mediate the function of AR in the ovarian granulosa cells of patients with PCOS. Genes diseases (2021) 8(1):94–109. doi: 10.1016/j.gendis.2019.09.005 33569518 PMC7859457

[108] Mehls O Ritz E Gilli G Heinrich U. Role of hormonal disturbances in uremic growth failure. Contributions to nephrology (1986) 50:119–29. doi: 10.1159/000412993 3026726

[109] Xue T Zhao S Zhang H Tang T Zheng L Jing J. PPT1 regulation of HSP90α depalmitoylation participates in the pathogenesis of hyperandrogenism. iScience (2023) 26(3):106131. doi: 10.1016/j.isci.2023.106131 36879822 PMC9984558

[110] Ma Y Andrisse S Chen Y Childress S Xue P Wang Z. Androgen receptor in the ovary theca cells plays a critical role in androgen-induced reproductive dysfunction. Endocrinology (2017) 158(1):98–108. doi: 10.1210/en.2016-1608 27841936 PMC5412974

[111] Caldwell ASL Edwards MC Desai R Jimenez M Gilchrist RB Handelsman DJ. Neuroendocrine androgen action is a key extraovarian mediator in the development of polycystic ovary syndrome. Proc Natl Acad Sci USA (2017) 114(16):E3334–e3343. doi: 10.1073/pnas.1616467114 28320971 PMC5402450

[112] Cara AL Burger LL Beekly BG Allen SJ Henson EL Auchus RJ. Deletion of androgen receptor in lepRb cells improves estrous cycles in prenatally androgenized mice. Endocrinology (2023) 164:bqad015. doi: 10.1210/endocr/bqad015 36683455 PMC10091504

[113] Xiong T Rodriguez Paris V Edwards MC Hu Y Cochran BJ Rye KA. Androgen signaling in adipose tissue, but less likely skeletal muscle, mediates development of metabolic traits in a PCOS mouse model. Am J Physiol Endocrinol Metab (2022) 323(2):E145–e158. doi: 10.1152/ajpendo.00418.2021 35658542

[114] Feng M Divall S Jones D Ubba V Fu X Yang L. Comparison of reproductive function between normal and hyperandrogenemia conditions in female mice with deletion of hepatic androgen receptor. Front endocrinol (2022) 13:868572. doi: 10.3389/fendo.2022.868572 PMC9218244 35757434

[115] Aflatounian A Edwards MC Rodriguez Paris V Bertoldo MJ Desai R Gilchrist RB. Androgen signaling pathways driving reproductive and metabolic phenotypes in a PCOS mouse model. J endocrinol (2020) 245(3):381–95. doi: 10.1530/joe-19-0530 32229702

[116] Coyle CS Prescott M Handelsman DJ Walters KA Campbell RE. Chronic androgen excess in female mice does not impact luteinizing hormone pulse frequency or putative GABAergic inputs to GnRH neurons. J neuroendocrinol (2022) 34(4):e13110. doi: 10.1111/jne.13110 35267218 PMC9286661

[117] Azziz R. PCOS: Animal models for PCOS - not the real thing. Nat Rev Endocrinol (2017) 13(7):382–4. doi: 10.1038/nrendo.2017.57 28474686

[118] Pei Y Risal S Jiang H Lu H Lindgren E Stener-Victorin E. Transcriptomic survey of key reproductive and metabolic tissues in mouse models of polycystic ovary syndrome. Commun Biol (2023) 6(1):69. doi: 10.1038/s42003-022-04362-0 36653487 PMC9849269

[119] Díaz M de Zegher F Ibáñez L. Circulating follistatin concentrations in adolescent PCOS: Divergent effects of randomized treatments. Front endocrinol (2023) 14:1125569. doi: 10.3389/fendo.2023.1125569 PMC9947640 36843579

[120] Yousuf SD Ganie MA Urwat U Andrabi SM Zargar MA Dar MA. Oral contraceptive pill (OCP) treatment alters the gene expression of intercellular adhesion molecule-1 (ICAM-1), tumor necrosis factor-α (TNF-α), monocyte chemoattractant protein-1 (MCP-1) and plasminogen activator inhibitor-1 (PAI-1) in polycystic ovary syndrome (PCOS) women compared to drug-naive PCOS women. BMC women's Health (2023) 23(1):68. doi: 10.1186/s12905-023-02187-5 36793022 PMC9933286

[121] Forslund M Melin J Alesi S Piltonen T Romualdi D Tay CT. Different kinds of oral contraceptive pills in polycystic ovary syndrome: a systematic review and meta-analysis. Eur J endocrinol (2023) 189(1):S1–s16. doi: 10.1093/ejendo/lvad082 37440702

[122] Bashir R Asrar MM Shah IA Wani IA Ganie MA. Do Pleiotropic Effects of Spironolactone in Women with PCOS Make it More than an Anti-androgen? Evidence from a Systematic Review and Meta-analysis. Curr Pharm design (2023) 29(19):1486–96. doi: 10.2174/1381612829666230331093912 36999713

[123] Wright C O'Day P Alyamani M Sharifi N Auchus RJ. Abiraterone acetate treatment lowers 11-oxygenated androgens. Eur J endocrinol (2020) 182(4):413–21. doi: 10.1530/eje-19-0905 PMC7096060 32045360

[124] Luo ED Jiang HM Chen W Wang Y Tang M Guo WM. Advancements in lead therapeutic phytochemicals polycystic ovary syndrome: A review. Front Pharmacol (2022) 13:1065243. doi: 10.3389/fphar.2022.1065243 36699064 PMC9868606

[125] Rana S Hussain L Saleem U Asif M Lodhi AH Barkat MQ. Dose dependent effects of aqueous extract of garcinia cambogia desr. Against letrozole induced polycystic ovarian syndrome in female adult rats with possible mechanisms exploration. Dose-response Publ Int Hormesis Society (2023) 21(2):15593258231169381. doi: 10.1177/15593258231169381 PMC10103256 37063342

[126] Rani R Chitme HR Kukreti N Pant P Abdel-Wahab BA Khateeb MM. Regulation of insulin resistance, lipid profile and glucose metabolism associated with polycystic ovary syndrome by tinospora cordifolia. Nutrients (2023) 15(10):2238. doi: 10.3390/nu15102238 37242122 PMC10221073

[127] Bizzarri M Monti N Piombarolo A Angeloni A Verna R. Myo-inositol and D-chiro-inositol as modulators of ovary steroidogenesis: A narrative review. Nutrients (2023) 15(8):1875. doi: 10.3390/nu15081875 37111094 PMC10145676

[128] Fedeli V Catizone A Querqui A Unfer V Bizzarri M. The role of inositols in the hyperandrogenic phenotypes of PCOS: A re-reading of larner's results. Int J Mol Sci (2023) 24(7):6296. doi: 10.3390/ijms24076296 37047265 PMC10093919

[129] Morgante G Darino I Spanò A Luisi S Luddi A Piomboni P. PCOS physiopathology and vitamin D deficiency: biological insights and perspectives for treatment. J Clin Med (2022) 11(15):4509. doi: 10.3390/jcm11154509 35956124 PMC9369478

[130] Ferro C Florindo HF Santos HA. Selenium nanoparticles for biomedical applications: from development and characterization to therapeutics. Advanced healthcare materials (2021) 10(16):e2100598. doi: 10.1002/adhm.202100598 34121366

[131] Abdallah ABE El-Ghannam MA Hasan AA Mohammad LG Mesalam NM Alsayed RM. Selenium nanoparticles modulate steroidogenesis-related genes and improve ovarian functions via regulating androgen receptors expression in polycystic ovary syndrome rat model. Biol Trace element Res (2023) 201:5721–5733. doi: 10.1007/s12011-023-03616-0 PMC10620277 36922476

[132] Butt MA Shafique HM Mustafa M Moghul NB Munir A Shamas U. Therapeutic potential of selenium nanoparticles on letrozole-induced polycystic ovarian syndrome in female wistar rats. Biol Trace element Res (2023) 201(11):5213–29. doi: 10.1007/s12011-023-03579-2 36694071

[133] Rabah HM Mohamed DA Mariah RA Abd El-Khalik SR Khattab HA AbuoHashish NA. Novel insights into the synergistic effects of selenium nanoparticles and metformin treatment of letrozole - induced polycystic ovarian syndrome: targeting PI3K/Akt signalling pathway, redox status and mitochondrial dysfunction in ovarian tissue. Redox Rep Commun Free Radical Res (2023) 28(1):2160569. doi: 10.1080/13510002.2022.2160569 PMC9870018 36661246

[134] Chu W Li S Geng X Wang D Zhai J Lu G. Long-term environmental exposure of darkness induces hyperandrogenism in PCOS via melatonin receptor 1A and aromatase reduction. Front Cell Dev Biol (2022) 10:954186. doi: 10.3389/fcell.2022.954186 36353509 PMC9639332

[135] Johnson BS Krishna MB Padmanabhan RA Pillai SM Jayakrishnan K Laloraya M. Derailed peripheral circadian genes in polycystic ovary syndrome patients alters peripheral conversion of androgens synthesis. Hum Reprod (Oxford England) (2022) 37(8):1835–55. doi: 10.1093/humrep/deac139 35728080

[136] Li S Zhai J Chu W Geng X Chen ZJ Du Y. Altered circadian clock as a novel therapeutic target for constant darkness-induced insulin resistance and hyperandrogenism of polycystic ovary syndrome. Trans Res J Lab Clin Med (2020) 219:13–29. doi: 10.1016/j.trsl.2020.02.003 32119846

[137] Roy S Abudu A Salinas I Sinha N Cline-Fedewa H Yaw AM. Androgen-mediated perturbation of the hepatic circadian system through epigenetic modulation promotes NAFLD in PCOS mice. Endocrinology (2022) 163(10):bqac127. doi: 10.1210/endocr/bqac127 35933634 PMC9419696

[138] Chen WH Huang QY Wang ZY Zhuang XX Lin S Shi QY. Therapeutic potential of exosomes/miRNAs in polycystic ovary syndrome induced by the alteration of circadian rhythms. Front endocrinol (2022) 13:918805. doi: 10.3389/fendo.2022.918805 PMC9709483 36465652

[139] Jamilian M Foroozanfard F Mirhosseini N Kavossian E Aghadavod E Bahmani F. Effects of melatonin supplementation on hormonal, inflammatory, genetic, and oxidative stress parameters in women with polycystic ovary syndrome. Front endocrinol (2019) 10:273. doi: 10.3389/fendo.2019.00273 PMC6527800 31139144

[140] Sucquart IE Nagarkar R Edwards MC Rodriguez Paris V Aflatounian A Bertoldo MJ. Neurokinin 3 receptor antagonism ameliorates key metabolic features in a hyperandrogenic PCOS mouse model. Endocrinol (2021) 162(5):bqab020. doi: 10.1210/endocr/bqab020 33522579

[141] Guo F Fernando T Zhu X Shi Y. The overexpression of neurokinin B-neurokinin 3 receptor system exerts direct effects on the ovary under PCOS-like conditions to interfere with mitochondrial function. Am J Reprod Immunol (New York NY 1989) (2023) 89(3):e13663. doi: 10.1111/aji.13663 36453600

[142] Chen WH Shi YC Huang QY Chen JM Wang ZY Lin S. Potential for NPY receptor-related therapies for polycystic ovary syndrome: an updated review. Hormones (Athens Greece) (2023) 22(3):441–51. doi: 10.1007/s42000-023-00460-8 PMC10449684 37452264

[143] Chugh RM Park HS El Andaloussi A Elsharoud A Esfandyari S Ulin M. Mesenchymal stem cell therapy ameliorates metabolic dysfunction and restores fertility in a PCOS mouse model through interleukin-10. Stem Cell Res Ther (2021) 12(1):388. doi: 10.1186/s13287-021-02472-w 34233746 PMC8261924

[144] Chugh RM Park HS Esfandyari S Elsharoud A Ulin M Al-Hendy A. Mesenchymal stem cell-conditioned media regulate steroidogenesis and inhibit androgen secretion in a PCOS cell model via BMP-2. Int J Mol Sci (2021) 22(17):9184. doi: 10.3390/ijms22179184 34502090 PMC8431467

[145] Park HS Chugh RM Pergande MR Cetin E Siblini H Esfandyari S. Non-cytokine protein profile of the mesenchymal stem cell secretome that regulates the androgen production pathway. Int J Mol Sci (2022) 23(9):4633. doi: 10.3390/ijms23094633 35563028 PMC9101816

[146] Altinkilic EM du Toit T Sakin Ö Attar R Groessl M Flück CE. The serum steroid signature of PCOS hints at the involvement of novel pathways for excess androgen biosynthesis. J Steroid Biochem Mol Biol (2023) 233:106366. doi: 10.1016/j.jsbmb.2023.106366 37499841

